# Isolation and identification of specific *Enterococcus faecalis* phage C-3 and G21-7 against Avian pathogenic *Escherichia coli* and its application to one-day-old geese

**DOI:** 10.3389/fmicb.2024.1385860

**Published:** 2024-06-19

**Authors:** Tianli Wang, Ling Zhang, Yi Zhang, Panpan Tong, Wanpeng Ma, Yan Wang, Yifan Liu, Zhanqiang Su

**Affiliations:** ^1^College of Veterinary Medicine, Xinjiang Agricultural University, Xinjiang, China; ^2^Xinjiang Key Laboratory of Herbivore Drug Research and Creation, College of Veterinary Medicine, Xinjiang Agricultural University, Xinjiang, China

**Keywords:** *Enterococcus faecalis* phage, virulent phage, genus *Saphexavirus*, Avian pathogenic *Escherichia coli*, one-day-old geese

## Abstract

Colibacillosis caused by Avian pathogenic *Escherichia coli* (APEC), including peritonitis, respiratory tract inflammation and ovaritis, is recognized as one of the most common and economically destructive bacterial diseases in poultry worldwide. In this study, the characteristics and inhibitory potential of phages were investigated by double-layer plate method, transmission electron microscopy, whole genome sequencing, bioinformatics analysis and animal experiments. The results showed that phages C-3 and G21-7 isolated from sewage around goose farms infected multiple O serogroups (O1, O2, O18, O78, O157, O26, O145, O178, O103 and O104) *Escherichia coli* (*E.coli*) with a multiplicity of infection (MOI) of 10 and 1, respectively. According to the one-step growth curve, the incubation time of both bacteriophage C-3 and G21-7 was 10 min. Sensitivity tests confirmed that C-3 and G21-6 are stable at 4 to 50 °C and pH in the range of 4 to 11. Based on morphological and phylogenetic analysis, phages C-3 and G21-7 belong to *Enterococcus faecalis (E. faecalis)* phage species of the genus *Saphexavirus* of *Herelleviridae* family. According to genomic analysis, phage C-3 and G21-7 were 58,097 bp and 57,339 bp in size, respectively, with G+C content of 39.91% and 39.99%, encoding proteins of 97 CDS (105 to 3,993 bp) and 96 CDS (105 to 3,993 bp), and both contained 2 tRNAs. Both phages contained two tail proteins and holin-endolysin system coding genes, and neither carried resistance genes nor virulence factors. Phage mixture has a good safety profile and has shown good survival probability and feed efficiency in both treatment and prophylaxis experiments with one-day-old goslings. These results suggest that phage C-3 and G21-7 can be used as potential antimicrobials for the prevention and treatment of APEC.

## 1 Introduction

Avian pathogenic *Escherichia coli* (APEC) is an important Extraintestinal pathogenic *E. coli* (ExPEC), which mainly causes peritonitis, respiratory inflammation (bronchitis, pneumonia, etc.), ovaritis, arthritis, balloon inflammation, cellulitis, head swelling syndrome and other local infections and systemic infections (Newman et al., 2021). These colibacillosis causes high morbidity and mortality in poultry, reduces meat and egg production, affects fertility, and increases carcass scrap rate at slaughter (Joseph et al., 2023). According to reports, APEC caused losses of 3.7 million euros, US$1.0969 billion and US$40 million to India, the Netherlands and the United States, respectively (De Brito et al., 2003; Landman and Van Eck, 2015; Wibisono et al., 2018). Disease caused by APEC is recognized globally as one of the most common and economically devastating bacterial diseases in poultry (Ghunaim et al., 2014). Currently, the main O-antigen serotypes associated with it include O1, O2, O18 and O78, accounting for 50% of APEC issues (Wang et al., 2014; Kathayat et al., 2021; Mehat et al., 2021). Our previous epidemiological research showed that in goose farms in China’s Xinjiang, 47.83% of APEC belonged to O1, O2, O18, and O78 (Cheng et al., 2022). In addition, these serotypes share the same highly pathogenic clonal group with neonatal meningitis, urinary tract infection, and sepsis, indicating that these APECs have posed potential zoonotic risks (Hu J. et al., 2022). A recent report suggested that O145 may also be becoming a major serogroup in APEC (Wang Z. et al., 2022). *E. coli* serogroup O145, O157, O103, O26, O104, and O178 have been implicated in human hemorrhagic colitis or hemolytic uremic syndrome (Frank et al., 2011; Miko et al., 2014; Ludwig et al., 2020). In particular, O157:H7 can cause serious gastrointestinal infections in children, and antibiotic treatment increases the risk of HUS (Wong et al., 2000). However, antibiotics are the global consensus for the prevention and treatment of bacterial diseases. But, due to the long-term irrational use of antibiotics and the horizontal transfer of drug-resistant genes, as well as the increased risk of serious diseases through antibiotic treatment, the prevention and control of bacterial diseases have brought greater challenges (Wong et al., 2000; Duan et al., 2021). According to hundreds of scholars’ statistics on the number of drug resistance-related deaths and disability-adjusted life years in 204 countries and regions in 2019, 49.5 million people died from bacterial resistance and 1.92 million people had a disability-adjusted life year affected (Murray et al., 2022). Therefore, bacterial antibiotic resistance has become one of the major public health security threats in the 21st century (Qiao et al., 2018).

APEC is already resistant to many antibiotics in the world and carries multiple resistance genes. They are resistant to chloramphenicol, tetracyclines, sulfonamides, aminoglycosides, fluoroquinolones and ß-lactam antibiotics, among others (Nhung et al., 2017). Of particular concern is the high APEC resistance to polymyxin found in many countries and regions. Polymyxin resistance of 70.1% was detected in isolates in Pakistan (Saeed et al., 2023). In the coastal regions of China, 42.1% of isolated strains show resistance to polymyxin and carry the mcr-1 gene (Hu Z. et al., 2022). In Vietnam, 8.7% of isolated strains are resistant to polymyxin, among which 85% carry the mcr-1 gene (Vounba et al., 2019). Additionally, all polymyxin-resistant bacteria exhibit a multiple drug resistance (MDR) phenotype (Vounba et al., 2019). In recent years, with the moderate growth in demand for goose and duck meat in Asia, countries such as Hungary and China, which are major exporters of these two meats, are also facing challenges in the prevention and control of APEC in the goose industry^1^. Studies have shown that APEC isolates from Hungarian geese are mainly of the O145 serogroup and are resistant to amoxicillin, ampicillin, ciprofloxacin and other drugs (Adorján et al., 2021); APEC geese in Henan, China are resistant to 5 to 12 levels of MDR (Zhang et al., 2023). In addition, a Chinese study found that goose-derived APEC is resistant to polymyxin and carries the mcr-1 gene, which is particularly similar to APEC strains derived from humans and birds (Hu Z. et al., 2022). Antibiotic-resistant APEC not only contributes to the failure of treatment of avian colibacillosis, but may also be the source of MDR bacteria or genes that pose human health risks (Hu J. et al., 2022). Therefore, there is an urgent need for a non-antibiotic alternative drug to treat avian colibacillosis caused by APEC.

Bacteriophages, as natural killers of bacteria, are considered a promising alternative to antibiotics for treating bacterial diseases (Soontarach et al., 2022). Bacteriophages are particularly toxic to resistant bacteria compared to conventional antibiotics (Aslam et al., 2019). In addition, phages typically recognize specific receptors on bacterial cell membranes, do not affect human or animal cells, and have few side effects on eukaryotic hosts (Sulakvelidze et al., 2001). At present, several phages have been reported to have lytic effect on APEC, and *in vivo* experiments have also shown that they have perfect protective effect on broilers (Wang L. et al., 2022; Tang et al., 2023; Yao et al., 2023). Most *Enterococcus faecium* (*E. faecalis*) phages can only lyse *E. faecalis*, and a few can lyse *Enterococcus faecium* (*E. faecium*). However, the effect of *E. faecalis* phage on APEC has not been reported. In this study, two strains of *E. faecalis* phage were isolated and their biological characteristics and genome sequence were analyzed. The therapeutic potential of phages was evaluated by animal experiments on goslings. In this study, we aimed to discover a phage that could be used as an alternative to antibiotics.

## 2 Materials and methods

### 2.1 Animals

Healthy 1-day-old goslings are provided by Xinjiang Kunlun Luyuan Goose Breeding Technology Co., Ltd. The experiments conducted in this study strictly complied with the Regulations on the Management of Laboratory Animals formulated by the State Science and Technology Commission of the People’s Republic of China and the Guiding Opinions on the Treatment of Laboratory Animals promulgated by the Ministry of Science and Technology of the People’s Republic of China, and were approved by the Experimental Animal Welfare Ethics Committee of Xinjiang Agricultural University (Ethical number: 2023015). Each group was raised separately in a wire mesh isolator equipped with a water fountain, a feeder, and a heating lamp. The room where the isolator is located is equipped with ventilation fans to allow efficient air circulation.

### 2.2 Samples and bacterial strains

The bacteriophage isolation samples were collected from sewage near goose farm in Xinjiang. A total of 72 strains of *Escherichia coli* (*E.coli*) from different sources (goose, cattle, sheep, camels, pigeon, human), different serogroup (O1, O2, O18, O78, O157, O26, O145, O178, O103 and O104) and all drug resistance (Supplementary Table S1) in our laboratory were selected. And a total of 221 strains of *Enterococcus faecalis* (*E. faecalis*) from different sources (cattle, pigeon, camel, goose, pig, dog) in our laboratory were selected (Supplementary Table S13). All strains were cultured at 37°C in Luria-Bertani (LB, Hopebio, China) and stored at −80°C in LB medium containing 30% glycerol.

### 2.3 Phage isolation and purification

According to previous reports, phages were isolated and purified by double-layer plate method (Chen et al., 2022). The sewage is centrifuged at 25°C at 5,000 × g for 10 min, and then filtered through a 0.45 μm filter (Merck Millipore, Germany) to remove impurities, and then filtered through a 0.22 μm filter (Merck Millipore, Germany) to remove macromolecular substances such as bacteria. 200 μL filtrate was mixed with 100 μL bacterial solution (OD_600_ = 0.6 ~ 0.8) in 4.7 mL LB medium containing 0.5% agar and the mixture was covered on LB agar plate (1.5% agar). The plate was incubated in an incubator at 37°C inverted for 10 h, and then transparent plaque was selected for further purification. After five repeated purification steps of single plaque, pure phage strains were obtained and stored at 4°C for further analysis.

### 2.4 Host-rang evaluation

The host range of bacteriophages in the Supplementary Table S1 strain (goose origin) was determined by spot test and double-layer agar plate method (Chen et al., 2022), and the bacteriophages with plaque against *E. coli* O1, O2, O18 and O78 were named C-3 and G21-7 (Supplementary Table S2). These two bacteriophages have been stored in the General Microbiology Center of China Microbiological Culture Preservation Management Committee (CGMCC) with the preservation numbers CGMCC No.45673 and CGMCC No.45674. C-3 and G21-7 further determined the host range of the Supplementary Table S1 strains (other than those of goose origin), including 61 *E. coli* strains with different serogroups.

### 2.5 Transmission electron microscopy

Phage particles were observed by ht7700 (HITACHI, Japan) transmission electron microscope. The purified bacteriophage particles (×10^10^ pfu mL^−1^) were dropped on a glass slide of 200 mesh copper film, and were negatively stained with phosphotungstate (PTA, 2% w/v). After natural drying for 20–30 min, the samples were observed at an accelerated voltage of 80 kV.

### 2.6 Growth characteristics: the optimal MOI and one-step experiments

Phage cultures were added to *E.coli* cultures with a concentration of ×10^10^ CFU·mL^−1^ at different Multiplicity of infection (MOIs) (1,000, 100, 10, 1, 0.1 and 0.01) to determine the optimal MOI. After incubation at 37°C for 12 h, the phagetiters were determined using the double-layer method. The experiments were repeated in triplicates. The MOI which produced the highest phage titer was identified to be the optimal MOI (Chen et al., 2022).

According to the method of Chen et al., the one-step growth curve of phage was plotted (Chen et al., 2022). To put it simply, the phage with the optimal MOI was co-cultured with bacteria (×10^10^ CFU·mL^−1^) for 10 min, so that the phage was fully adsorbed. The unadsorbed phages were removed by centrifugation, and then the precipitation was suspended in 10 mL fresh LB medium for culture at 150 r/min at 37°C. The phage titer was measured by double AGAR method every 10 min. The experiment was repeated three times.

### 2.7 Sensitivity of temperature and pH

In order to measure the temperature sensitivity of phage, 500 μL phage suspension was immersed in constant temperature water bath for 30 min and 60 min at different temperatures (40, 50, 60, 70, 80°C). 100 μL phage suspension was added to 900 μL LB broth with different pH (4, 5, 6, 7, 8, 9, 10, 11) values and incubated at 37°C for 10 min. Immediately afterwards, phage titers were assessed using the double-plate method. These experiments were repeated three times.

### 2.8 DNA extraction and genome sequencing

The phage DNA genome was extracted using HiPure Viral DNA Mini kit D3191-02 (Magen, China). Complete genome sequencing of phage was carried out using the Illumina HiSeq system (Illumina, San Diego, CA, United States, RRID:SCR_016386). The reads were QC and then assembled using velvet, gap filled with SSPACE and GapFiller (Zerbino and Birney, 2008; Zerbino et al., 2009; Boetzer et al., 2011; Boetzer and Pirovano, 2012; Hunt et al., 2014). The assembled whole genome sequence has been uploaded to NCBI’s GenBank database, and GenBank accession is PP858896 and PP848412, respectively.

### 2.9 Annotation of the genome

The phage genome was annotated using Phigaro 2.3.0 (Starikova et al., 2020), and VirSorter 2.2.4 (Starikova et al., 2020), and the full genome was mapped using Proksee 1.2.0^2^. Meanwhile, the genome was annotated in more detail using Bakta v1.8.2 (Schwengers et al., 2021) and PhageScope^3^ (Wang et al., 2024). PhageScope online website was used to predict Anti-CRISPR, Antimicrobial Resistance Gene and Virulent Factor. PhaTYP^4^, a computational method used for phage lifestyle classification (Shang et al., 2023). PhaGCN^5^, a tool for classifying phages (based on the International Committee on Taxonomy of Viruses, ICTV) (Shang et al., 2021). CHERRY^6^, a computational method used to predict the host of a phage (Shang and Sun, 2022). A BLASTn search against the NCBI database identified C-3/G21-7-like phages, and the sequence homology was aligned using GBKviz 1.2.0^7^.

### 2.10 Phylogenetic analysis

Based on the phage’s whole genome sequence and terminase large subunit nucleotide sequence, Mega 11 (RRID:SCR_023017) performed phylogenetic analyses using the Neighbor-joining method with 1,000 bootstrap replicates and visualized using Chiplot^8^ (Xie et al., 2023). Phages were compared with 10 phage nucleotide sequence on the NCBI website. In addition, OAT is used to assess ANI values to assess the genetic relationships of species at the genomic level (Lee et al., 2016). According to ICTV Bacterial and Archaeal Viruses Subcommittee (BASV) regulations, the difference in nucleotide sequence between two viruses belonging to the same species should be less than 5%. Therefore, the classification of phage genera and species takes into account more than 73.98 and 95% values (Jain et al., 2018; Barco et al., 2020).

### 2.11 Prediction of tail protein conserved domain and motif

A first step in the interaction of phage interaction with bacteria is usually associated with receptor-binding proteins on the tail fibers and/or tail spikes (Domingues et al., 2021). Based on the phage’s tail protein sequence, Mega 11 performed phylogenetic analyses using the Neighbor-joining (N-J) method with 1,000 bootstrap replicates and visualized using Chiplot. Tail protein sequence were compared with 8 phage amino acid sequences on the NCBI website. The Conserved Domain Database (CDD) of NCBI was used to predict conserved domains in batches^9^. Use MEME Suite 5.5.5 to predict motifs in batches^10^.

### 2.12 Safety test of phage mixtures *in vivo*

The bacteriophage mixture of C-3 and G21-7 was prepared in a ratio of 1:1 according to the titer corresponding to the optimal MOI, and safety was evaluated by oral (mixture: drinking water = 1:100) and intraperitoneal injection (0.2 mL per goose) in 1-day-old geese (70 g ± 20 g). Daily feed intake, weekly body weight, and number of deaths were recorded for each group (Supplementary Table S9), and 21 day feed efficiency (Feed Efficiency = Increased weight/feed consumption) and survival were calculated (Kaplan–Meier analysis and log-rank test were used).

### 2.13 Evaluation of the efficacy of phage mixture and commercial antibiotics in the treatment or prevention of APEC infection *in vivo*

In order to verify whether phage mixture can replace antibiotic treatment, 1-day-old goslings (*n* = 300) were randomly divided into 6 groups, which were intrabitoneally injected with 0.02 mL of APEC (O1 serotype) of 1 × 10^7^ CFU/mL. Six hour later, each group was given phage mixture (for 3 days), Spectinomycin Hydrochloride and Lincomycin Hydrochloride Soluble Powder (SHLHSP, Ringpu, China), Tiamulin Fumarate Soluble Powder (TFSP, Ringpu, China), Compound Sulfamonomethoxine Sodium Soluble Powder (CSSSP, Shanxi Yi Kang Animal’s Pharmaceutcal, China), Doxycycline Hyclate Soluble Powder (DHSP, Aether Centre (Beijing) Biology, China) and Fubennikao Fen (FF, Aether Centre (Beijing) Biology, China), respectively. Antibiotics are taken orally in accordance with the minimum dosage and applicable days specified in the commercial instructions. In order to compare the effects of bacteriophage mixture and antibiotics on the prevention of APEC infection, 1-day-old goslings (*n* = 270) were randomly divided into 6 groups, and each group was given oral administration of bacteriophage mixture (1:100), Spectinomycin Hydrochloride and Lincomycin Hydrochloride Soluble Powder, Tiamulin Fumarate Soluble Powder, Compound Sulfamonomethoxine Sodium Soluble Powder, Doxycycline Hyclate Soluble Powder and Fubennikao Fen for 3 days. After intraperitoneal injection of 0.02 mL of 1 × 10^7^ CFU/mL of APEC (O1 serotype), observation was started for 21 days. Other goslings (*n* = 50) were injected with 0.02 mL of APEC (1 × 10^7^ CFU/mL) as a positive control group, and goslings (*n* = 32) were injected with 0.02 mL of pre-cooled PBS as a negative control group. Daily feed intake, weekly body weight, and number of deaths were recorded for each group (Supplementary Tables S10, S11), and 21 day feed efficiency (Feed Efficiency = Increased weight/feed consumption) and survival were calculated (Kaplan–Meier analysis and log-rank test were used).

### 2.14 Statistical analysis

SPSS Statistics 26 (RRID:SCR_016479) software was used to complete statistical analysis, and the test set and control set were compared by Mann–Whitney U test, *p* < 0.05 was considered statistically significant. Chiplot online website (see footnote 8) provides visualization (Xie et al., 2023). Kaplan–Meier analysis and log-rank test used two R packages (RRID:SCR_001905), survival and survminer, for visual analysis Graphpad 9.5 (RRID:SCR_002798) was used for Surival Cure comparison between two groups. Vector image composition using Adobe Illustrator 2024 software (RRID:SCR_010279).

## 3 Results

### 3.1 Isolation and identification of phages

**Figure 1:**
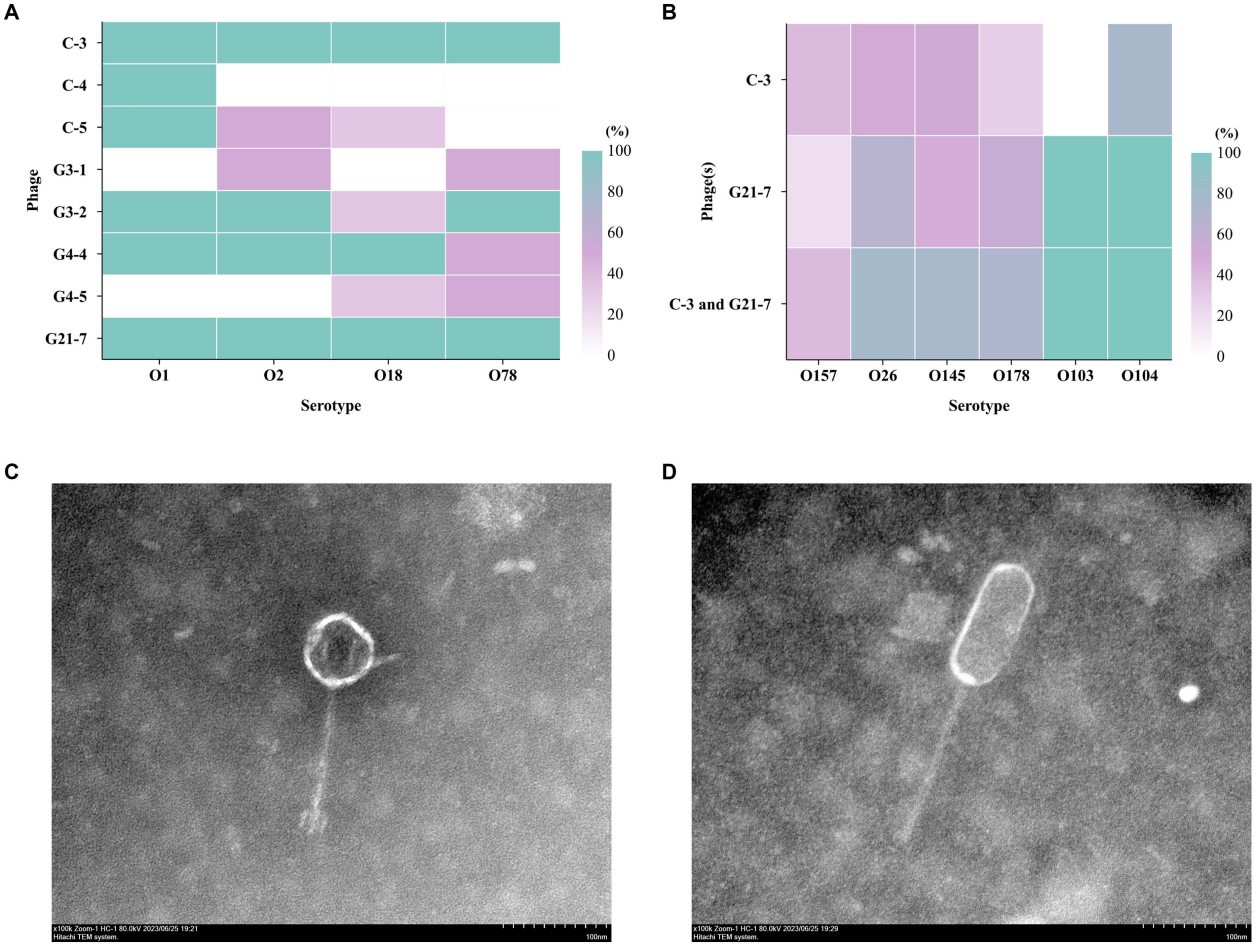
The host range of the phage. **(A)** Host spectrum of 8 bacteriophages against different serum groups of APEC; **(B)** Host profile of phage C-3 and C-5 against 61 strains of *E. coli* with different serogroups (different sources); Transmission electron micrographs (The bar indicates 100 nm); **(C)** C-3 was estimated approximately 75 (±1) nm between opposite apices and the head was connected to a long 135 (±1) nm tail; **(D)** G21-7 was estimated approximately 115 (±1) nm between opposite apices and the head was connected to a long 160 (±1) nm tail.

**Figure 2:**
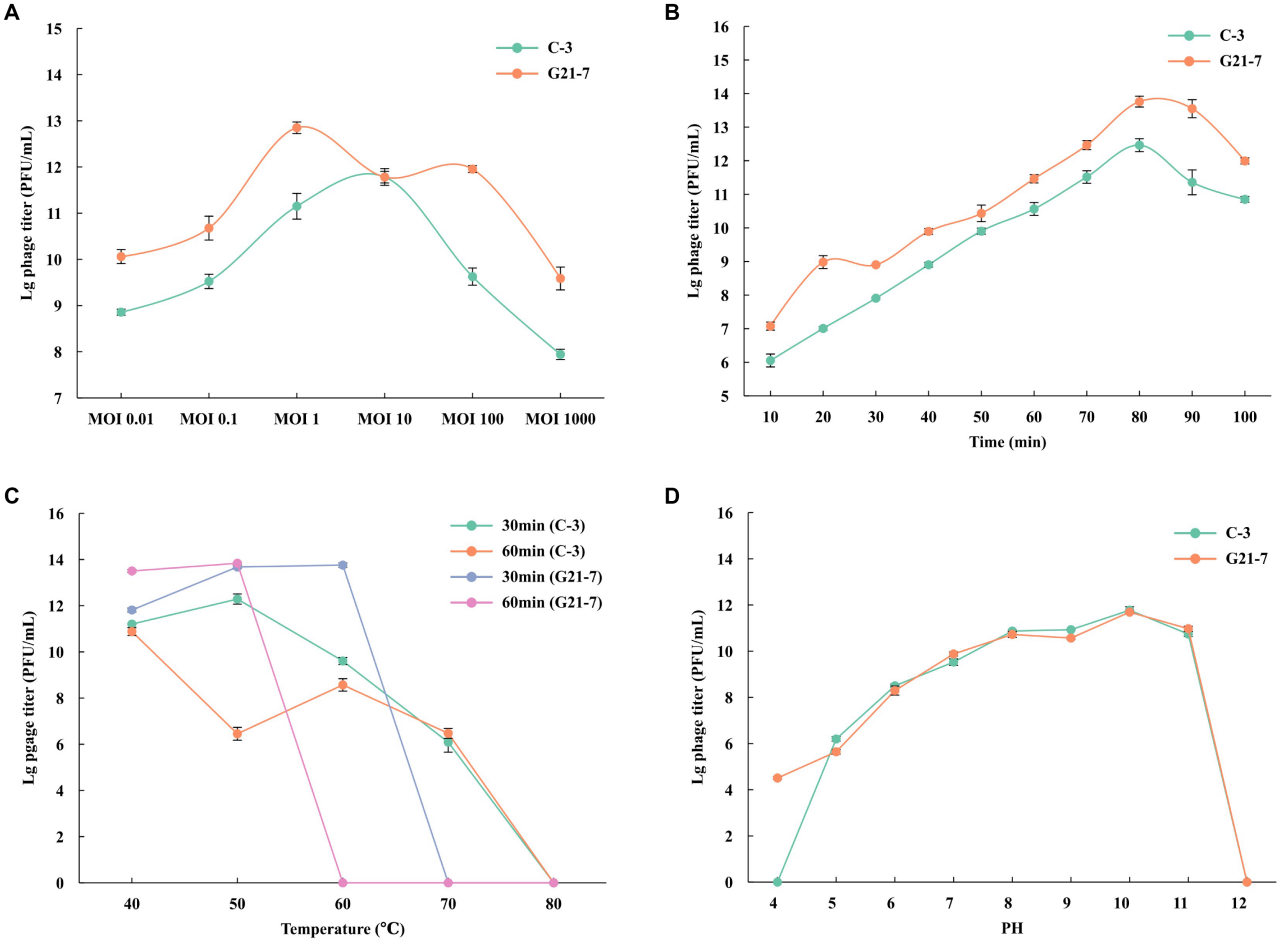
Growth characteristics: the optimal MOI and one-step experiments. **(A)** The Optimal Multiplicity of Infection (MOI) phage C-3/G21-7 was tested under different MOIs (1,000, 100, 10, 1, 0.1 and 0.01). When at an MOI of 10, phage C-3 produced highest phage titer; when at an MOI of 1, phage G21-7 produced highest phage titer. **(B)** The one-step growth curve of phage C-3/G21-7. Sensitivity of Temperature and PH. **(C)** Sensitivity of temperature: phage C-3/G21-7 suspension was incubated in different temperatures (40, 50, 60, 70, 80°C) for 30 min or 1 h. **(D)** Sensitivity of PH: phage C-3/G21-7 suspension was incubated in different PH conditions for 10 min. Phage survival rate = (titer after incubation)/(initial titer). The standard deviation is indicated by a vertical line.

**Figure 3:**
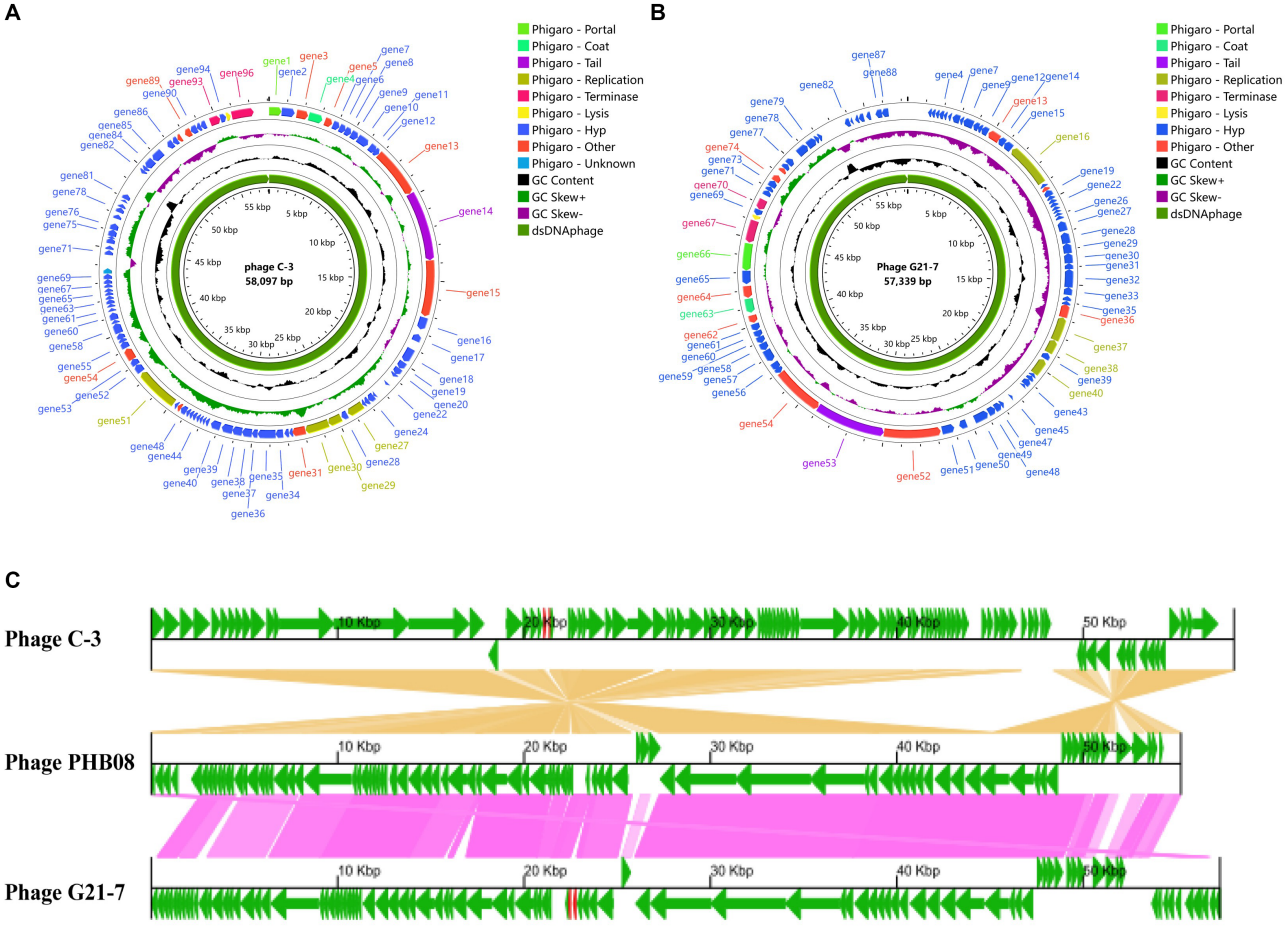
Genome analysis. **(A)** Phage C-3 genome map. **(B)** Phage G21-7 genome map. Both are drawn using Proksee. **(C)** After obtaining CDS annotations through Bakta, a collinearity analysis graph was drawn. Comparison of C-3 and G21-7 with the highly homologous phage PHB08 (MK570225, 55,244 bp). The genome encoding the prediction protein is shown in green, tRNA is shown in red.

Using different serotypes of APEC from geese as host bacteria, eight phages were isolated from sewage using double-layer agar method, namely C-3, C-4, C-5, G3-1, G3-2, G4-4, G4-5, G21-7 (Figure 1A and Supplementary Table S2). Among them, C-3 and G21-7 had lytic effect on 8 strains of APEC. Based on TEM (Figures 1C,D), C-3 has estimated approximately 75 (±1) nm between opposite apices, and the head was connected to a short 135 (±1) nm tail; G21-7 has estimated approximately 115 (±1) nm between opposite apices, and the head was connected to a short 160 (±1) nm tail. It was confirmed that C-3 and G21-7 belonged to order *Caudovirales* and were two different forms of phages.

### 3.2 Phages host range

Using spot test and double-layer agar plate test, Phages C-3, G21-7 and both were tested against 61 *E. coli* isolates of different serogroups and 221 *E. faecalis* isolates of different sources (Supplementary Tables S1, S13). Interestingly, in the spot test, C-3 and G21-7 were found to have different levels of infection against *E. coli* O157, O26, O145, O178, O103, O104, and the double-layer agar plate test had similar results (Figure 1B). The results showed that C-3 and G21-7 had extensive lytic activity against *E. coli* groups with potential to infect humans. Second, it was particularly shown that the combination of C-3 and G21-7 covers a larger host range than alone. However, C-3 and G21-7 were not found to have lytic activity against *Enterococcus faecalis*.

### 3.3 Growth characteristics: the optimal MOI and one-step experiments

Pnagus C-3 produced the highest titer at MOI = 10, indicating that MO1 = 10 was the best MOI for C-3; Phage G21-7 produced the highest titer at MOI = 1, indicating that MOI = 1 was the best MOI for G21-7 (Figure 2A and Supplementary Table S3). According to the one-step growth curve, Phages C-3 and G21-7 have an incubation period of about 10 min, After 30 min, it starts to steadily increase exponentially, and then the maximum phage titers produced at 80 min were 3.1 × 10^12^ PFU/mL and 6.0 × 10^13^ PFU/mL, respectively (Figure 2B and Supplementary Table S4).

### 3.4 Sensitivity of temperature and pH

The temperature sensitivity of phage C-3 was determined by incubation at 40, 50, 60, 70, 80°C for 30 min and 1 h. Phage C-3 was found to be active when incubated at temperatures between 40 and 60°C for 30 min or 1 h, with a high phage survival rate, a sharp decline at 70°C, and inactivation at ≥80°C. Phage G21-7 was found to be active and had a high survival rate when incubated between 40 and 60°C for 30 min, but then decreased sharply and became inactive when the temperature was ≥70°C. Survival rate was higher when incubated at temperatures between 40 and 50°C for 1 h, followed by a sharp decline, and inactivation was observed at temperatures ≥60°C (Figure 2C and Supplementary Table S5). The results showed that phage G21-7 was less sensitive to high temperature than C-3.

Phage C-3 is active in the pH4 to pH11 range, but completely loses infectiousness in acidic (pH4) and alkaline mediators (pH12). Phage G21-7 is active in the pH4 to pH11 range, but completely loses infectiousness in alkaline mediators (pH12; Figure 2D and Supplementary Table S6). A comparison shows that the acid resistance of G21-7 was stronger than that of C-3.

### 3.5 Genome analysis

Using Illumina whole genome sequencing, the phage C-3 genome was a double-stranded DNA phage (dsDNAphage) with a measured 58,097 bp, a GC content of 39.91%, 97 coding sequence (CDS, 105 to 3,993 bp in length) and 2 transfer RNA (tRNA). 95 genes were assigned to different Viral Orthologous Groups (VOG) by Phage Viral Orthologous Groups (POVG, Figure 3A and Supplementary Table S7), 18 of which were annotated as known functional VOG. Tail fibers protein (gene14) was predicted by Phigaro, Bakta and PhageScope, and another tail protein, Phage major tail protein 2 (gene10), was also predicted by PhageScope (Supplementary Table S7). These two proteins are key determinants of the phage’s host-specific and infective processes (Nobrega et al., 2018). Annotated genome sequence by Bakta, N-acetylmuramoyl-L-alanine amidase is present, which is reported to be an enzyme that hydrolyzes the peptidoglycan of bacterial cell walls (Supplementary Table S7) (García-Cano et al., 2019). It is usually used as endolysin and together with the holin protein that can destroy the cell membrane to form the Holin-Lysin system, which performs phage cleavage and progeny release for bacteria (Liu et al., 2022). In addition, chitinase, an enzyme that acts on the fungal cell wall, was found (Li et al., 2022). Secondly, no Anti-CRISPR proteins were found with PhageScope. However, transferase activity proteins (gene31, 40, 89) were found to be associated with the bacteriophage escape Restriction modification(R-M) system (Samson et al., 2013).

Phages G21-7 is a dsDNAphage with a genome measurement length of 57,339 bp, a GC content of 39.99%, 96 CDS (105 to 3,993 bp in length) and 2 tRNA. POVG assigned 88 genes to different VOG (Figure 3B and Supplementary Table S8), 19 of which were annotated as known functional VOG. Similar to phage C-3, tail protein structure (Phage tail protein, gene53; Phage major tail protein 2, gene57), N-acetylmuramoyl-L-alanine amidase and holin, and transferase activity protein (gene27, gene36, gene74) were predicted by different prediction software, and again did not have Anti-CRISPR (Supplementary Table S8). Two phages were predicted using PhageScope, neither of which contained resistance genes and virulent factor. In addition, PhaTYP predicted that phage C-3 and G21-7 were virulent, and PhaGCN predicted that both were *Herelleviridae*, hosted by *E. faecalis*. Meanwhile, the bacteriophage most closely related to C-3 and G21-7 was identified by online BLASTn using the NCBI database as virulent bacteriophage of *E. faecalis* of the *Herelleviridae*, called *Enterococcus* phage PHB08 (MK570225), which had 96.40% similarity and 89% query overage with C-3, 96.48% similarity and 88% query overage with G21-7. The GBKviz (Figure 3C) visual whole-genome linear comparison also shows that the three genomes are highly similar.

### 3.6 Phylogenetic analysis

**Figure 4:**
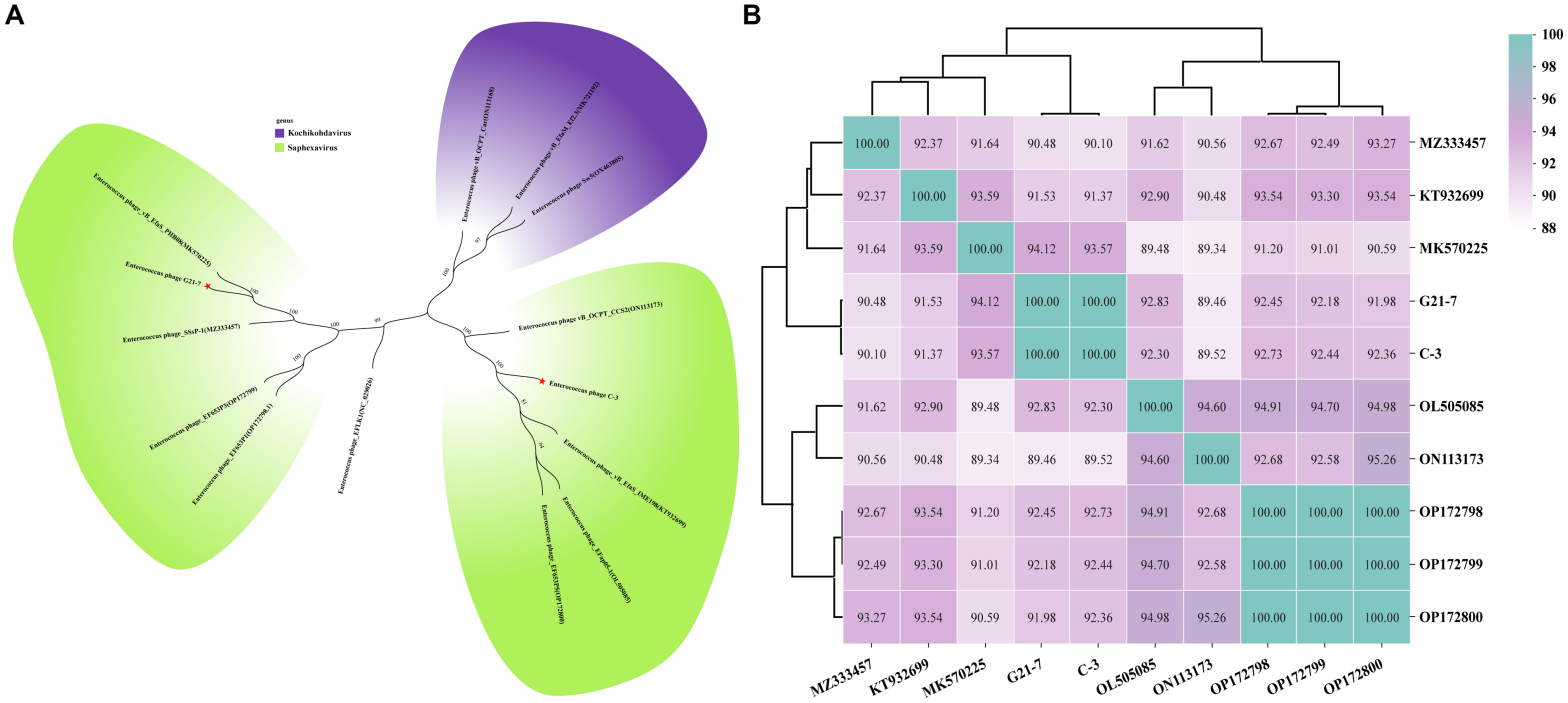
Phylogenetic analysis and ANI clustering. **(A)** Whole genome sequences of 12 phages from the NCBI website were compared. Purple indicates *Kochikohdavirus* genus (including separate clades) and green indicates *Saphexavirus* genus. Stars have been identified for two phages C-3 and G21-7 in this paper. **(B)** The ANI values between phage C-3 and G21-7 and *Saphexavirus* genomes were clustered by Ward’s linkage method.

Phylogenetic trees based on the whole genome sequence showed that both phage C-3 and G21-7 belonged to two clades of the genus *Saphexavirus*, indicating significant differences in the phylogenetic relationship between the two phages (Figure 4A). Further, the ANI values of phages C-3 and G21-7 and the above eight *Saphexavirus* phages were evaluated (Figure 4B). The results showed that both phages were >89.46% but <95%, indicating that both phages belonged to the new species of *Saphexavirus* genus of *Herelleviridae* family. Although the ANI values of C-3 and G21-7 were 100%, combined with phylogenetic and TEM results, the two phages belonged to the same species but different subspecies. Phylogenetic tree based on terminase large subunit nucleotide sequences showed that both phage C-3 and G21-7 belonged to same clade of species *Enterococcus faecalis* phages, this results were the same as those of whole genome sequence phylogenetic analysis and linear comparison.

### 3.7 Tail protein domain and motif prediction

**Figure 5:**
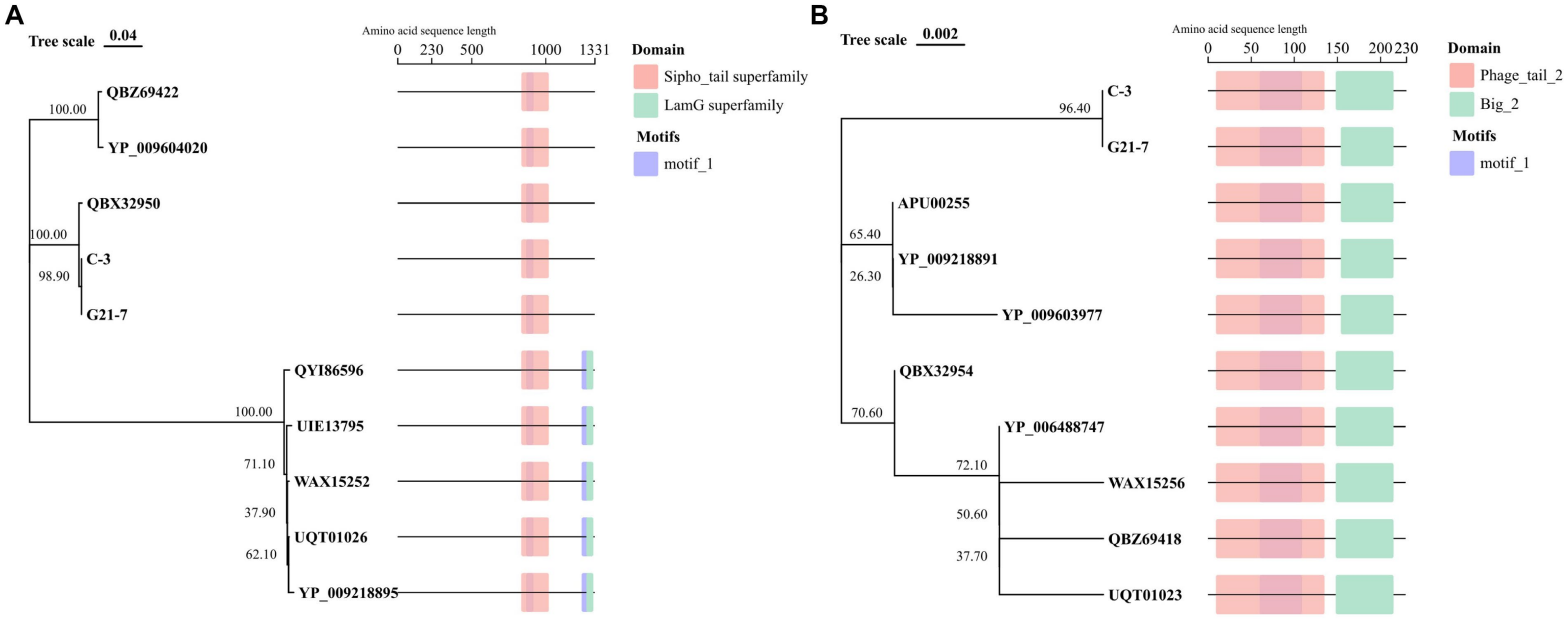
Tail protein sequence analysis. **(A)** Tail fibers protein of 8 phages from the NCBI website were compared with domain and motif structure analysis. **(B)** Phage major tail protein 2 of 8 phages from the NCBI website were compared with domain and motif structure analysis.

Two tail proteins were predicted in both C-3 and G21-7, and N-J evolutionary tree with conserved domains and motifs were constructed according to corresponding protein sequences. The results show that C-3 and G21-7 cluster with Phage PHB08 (QBX32950), and the tail fibers protein domain is 835 to 1,021, belonging to the Sipho_tail superfamily. Different from another group of *E. faecalis* Phage, it does not have the LamG superfamily domain located in 1,275 to 1,322. motif prediction results show that C-3 and G21-7 have a motif (868 to 917) that is contained in the Sipho_tail superfamily domain. Another analysis of the sequence of Phage major tail protein 2 showed that C-3 and G21-7 were clustered into a single cluster containing two domains, namely Phage_tail_2 (9 to 135) and Big_2 (148–215). A motif (60 to 109) is encountered in the Phage_tail_2 family domain (Figure 5).

### 3.8 Safety test of phage mixtures *in vivo*

**Figure 6:**
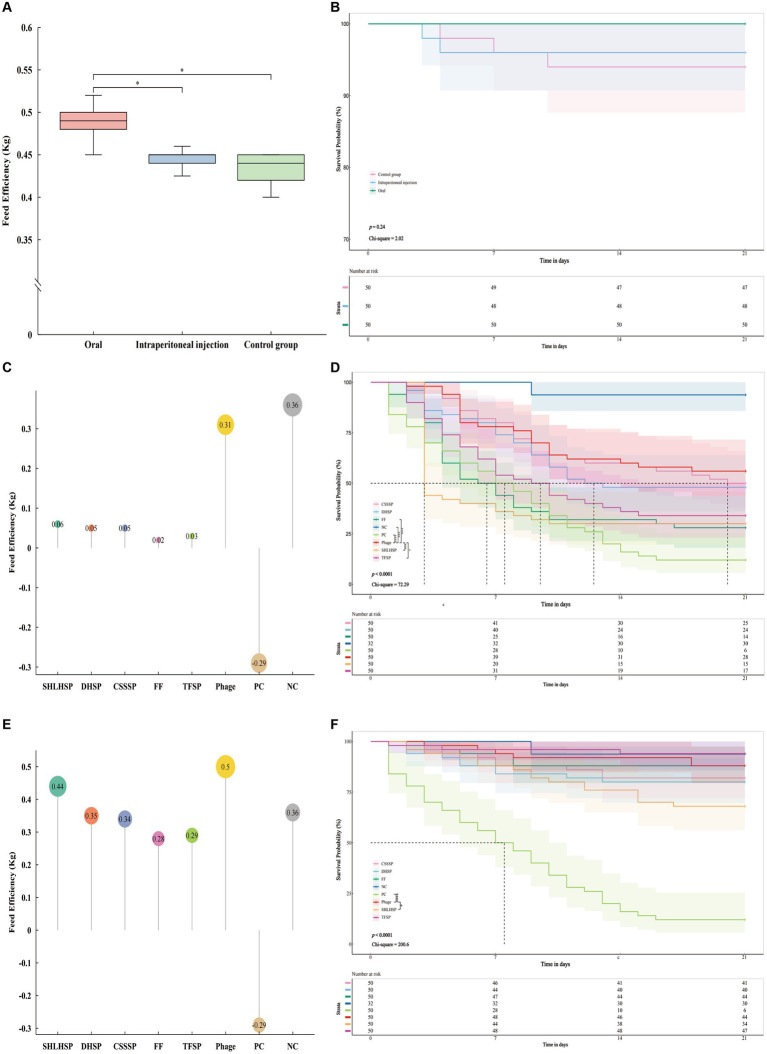
Safety test of phage mixtures *in vivo*, **(A)** feed efficiency (feed efficiency = Increased weight/feed consumption) was significantly different in the oral group compared with the intraperitoneal injection group (*p* < 0.05) and the control group (*p* < 0.05); **(B)** Kaplan–Meier analysis curves and log-rank test showed that *p* value = 0.24 and Chi-square = 2.02 between oral, intrabitoneal injection and control group showed no difference. Pairwise comparison showed no difference (*p* > 0.01, Supplementary Table S11). Treatment test *in vivo*, **(C)** feed efficiency of Phage (0.31 kg) was similar to that of the negative control group (0.36 kg) and higher than that of the other antibiotic groups; **(D)** Kaplan–Meier analysis curves and log-rank test showed that *p* value <0.0001 and Chi-square = 72.29 among all groups; The Log rank test was conducted between phage and other groups, respectively. The phage showed significant differences with TFSP, FF, SHLHSP, PC, and NC, but no differences with CSSSP and DHSP. Prevention test *in vivo*, **(E)** the phage group (0.5 kg) had the highest feed efficiency; **(F)** Kaplan–Meier analysis curves and log-rank test showed that *p* value <0.0001 and Chi-square = 200.6 among all groups; the Log rank test was conducted between phage and other groups respectively, phage showed significant differences with SHLHSP and PC, but no differences with other groups. Number of risk shows the number of geese that did not die for 21 days. TFSP, Tiamulin Fumarate Soluble Powder (Veterinary Drug Character (VDC) 020033008, Ringpu, China); FF, Fubennikao Fen (VDC 010122539, Aether Centre (Beijing) BIOLOGY, China); CSSSP, Compound Sulfamonomethoxine Sodium Soluble Powder (VDC 040266233, SHANXI YI KANG ANIMAL’S PHARMACEUTCAL, China); DHSP, Doxycycline Hyclate Soluble Powder (VDC 010126011, Aether Centre (Beijing) BIOLOGY, China); SHLHSP, Spectinomycin Hydrochloride and Lincomycin Hydrochloride Soluble Powder (VDC 20031339, Ringpu, China); PC, positive control; NC, negative control. “*” *p* < 0.05, “**” *p* < 0.01, “***” *p* < 0.001, and “****” *p* < 0.0001.

The 21 day feed efficiency of each group was measured by Wilcoxon signed-rank test (Figure 6A). The results showed that the oral group was significantly higher than the intrabitoneal injection group (*p* < 0.05) and the control group (*p* < 0.05). The average weight of each goose in the oral phage mixture group increased by 0.001 kg when feeding the same weight (1 kg) diet. Kaplan–Meier analysis curves and log-rank test showed no significant difference in overall survival between oral, intrabitoneal and control groups (*p* > 0.5, Chi-square = 2.838, Figure 6B). Further pairwise comparison showed no significant difference (Figure 6B and Supplementary Table S11). The feed efficiency and survival analysis showed that phage injection into geese by oral or intraperitoneal injection had little effect on animal safety. However, oral phages can improve animal body weight through feed efficiency reaction.

### 3.9 Evaluation of the efficacy of phage mixture and commercial antibiotics in the treatment or prevention of APEC infection *in vivo*

Based on the safety trial results, phage mixtures were selected to be administered orally to evaluate the efficacy of treatment and prevention of APEC infection in geese. The feed efficiency of phages and different antibiotics in treating geese infected with APEC within 21 days showed that the feed efficiency of phages group was 0.31 kg (Figure 6C), which was 5.17 times higher than that of SHLHSP group, DHSP and CSSSP groups, FF group 15.5 times, and TFSP group 10.33 times. Surprisingly, the feed efficiency of the phages group was only slightly lower than that of the negative control group (NCG, 0.36 kg). Kaplan–Meier analysis curves and log-rank test showed significant differences in survival among different groups (*p* < 0.0001, Chi-square = 72.29, Figure 6D). Further pairwise comparison between phages and other groups showed that there was no significant difference in survival rate between phages and CSSSP and DHSP groups (Supplementary Table S11), and the survival rate after 21 days was similar, which was 28, 25, and 24, respectively (Figure 6D). Surprisingly, phage survival rates were significantly higher than those of the other antibiotic groups (Supplementary Table S11). In addition, it was found that the survival rate of the bacteria group was significantly lower than that of the NCG (*p* < 0.001), but the Ratio = 8.651 and 95% CI of Ratio were 3.864 to 19.37, indicating that geese in the NCG were exposed to the risk of death (Supplementary Table S11).

Geese in the NCG were exposed to a risk of death through treatment trials, so the efficacy of bacteriophage and different antibiotics in preventing APEC infection in geese was further evaluated. The feed efficiency of geese in different groups was calculated within 21 days. The results showed that the feed efficiency of phage group was 0.5 kg (Figure 6E), which was 1.14 times higher than SHLHSP group, 1.43 times higher than DHSP group, 1.47 times higher than CSSSP group, 1.79 times higher than FF group and 1.72 times higher than TFSP group, respectively. Surprisingly, the feed efficiency of the phage group was 1.39 times higher than that of the NCG. Kaplan–Meier analysis curves and log-rank test showed significant differences in survival among different groups (*p* < 0.0001, Chi-square = 200.6, Figure 6F). Further pairwise comparison between phage and other groups showed that there was no significant difference in survival rates between phage and CSSSP, DHSP, FF and TFSP groups (Supplementary Table S11), and the survival rates after 21 days were similar, 44, 41, 40, 44, and 47, respectively (Figure 6D). The difference between SHLHSP and SHLHSP was significant, and it was significantly increased (Supplementary Table S11). Surprisingly, there was also no significant difference in mortality between the phage group and the NCG. The results showed that the effect of bacteriophages on the prevention of APEC infection was similar to, or even higher than, some antibiotics. Combined with feed efficiency and survival analysis, the use of phages not only promoted feed efficiency, but also reduced the risk of death of exposed geese in the NCG.

## 4 Discussion

In this study, phages C-3 and G21-7, which are infectious to different serotypes of *E.coli*, were isolated and identified by genomic and phylogenetic analysis as highly similar to *E. faecalis*. This phenomenon may be determined by the host specificity of phage tail proteins (Wang et al., 2021). Due to differences in the domain of tail proteins, the highly genomically similar *Shigella* phage SFPH2, *Citrobacter* phage SH4, and *Cronobacter* phage Dev2 have different host ranges (Yang et al., 2018). Interestingly, in order to explain the host differences between *E. faecalis* phage C-3 and G21-7, two tail proteins of both phages were analyzed in this study, and the Big_2 domain of tail protein 2 was able to bind to carbohydrate components Such as peptidoglycan or lipopolysaccharide (LPS) (Halaby and Mornon, 1998; Fraser et al., 2007). The nucleotide sequence of another tail fiber protein in both phages was clustered into the same branch as phage PHB08 (QBX32950), and the domains Sipho_tail superfamily and LamG superfamily were also identical (Yang et al., 2020). Genomic analysis of phages C-3 and G21-7 showed the ability to recognize both *E. coli* and *E. faecalis*. Unfortunately, due to the limited number of *E. faecalis* in our laboratory, it is not possible to verify the infective capacity of two phages in *E. faecalis*. In addition, another element of the phage’s ability to infect both strains of Gramella may be related to the catalytic domain (CD) and the C-terminal cell wall-binding domain (CBD) in endolysins (Oechslin et al., 2022). At present, phages with a broad host spectrum are mainly concentrated in Gram-negative bacteria. For example, Tequatrovirus EP01 could degrade *Salmonella Enteritidis* (*S. enteritidis*), *Yersinia pestis* (*Y. pestis*) could infect Escherichia, Shigella and Salmonella (Jin et al., 2022).

The host cleavage of most dsDNA phages after completion of the replication cycle is mainly related to holin proteins and N-acetylmuramoyl-L-alanine amidase. Holin protein first forms micron-scale pores in the bacterial inner membrane, and then releases endolysin into the periplasm to degrade peptidoglycans (Cahill and Young, 2019). N-acetylmuramoyl-L-alanine amidase involved in the degradation of peptidoglycan in bacterial cell wall and has the function of hydrolyzing the amide bond between N-acetylmuramoyl-L-alanine (García-Cano et al., 2019). This enzyme acts as an endolysin to form the holin-endolysin system with the holin predicted in this study, and the genes encoding this system are usually adjacent in the phage genome (Cahill and Young, 2019; Xi et al., 2019). However, the genes encoding the holin-endolysin system in this study are far apart in phages C-3 (gene15 and gene95) and G21-7 (gene51 and gene68). Other phages have similar phenomena, such as Klebsiella phage SH-KP2226 and *Aeromonas hydrophila* (*A. hydrophila*) phage PZL-Ah152 (Wu et al., 2019; Feng et al., 2022). Our team will conduct a detailed study of the holin-endolysin system for studying phages in the future to improve our understanding of the system in which Gram-positive phages can lyse Gram-negative bacteria phages contain resistance genes and virulence genes that may raise safety concerns in the development of therapeutic agents (Tang et al., 2023). Secondly, phage stability is also critical for phage vaccine transport and storage (Wang et al., 2022a). In this study, the two bacteriophages did not carry drug resistance genes and virulence genes, and had strong resistance to high temperature and acid and alkali, which could be used as good candidates for therapeutic agents. Oral bacteriophages can be transferred to various organs through systemic circulation and can regulate intestinal health without destroying intestinal flora, which is considered a reliable mode of drug delivery (Mendes et al., 2022; Zhao et al., 2022). It has been reported that the oral T4 phage treatment of 75 children with acute bacterial diarrhea safety analysis, liver, kidney and hematological function is normal (Sarker et al., 2016). Oral of *E. faecalis* phage did not cause damage to mice with ethanol-induced liver disease and inhibited *E. faecalis* (Mendes et al., 2022. In the safety evaluation of this study, oral phage did not cause the death of geese, and it was unexpectedly found that the feed efficiency of oral phage on geese was significantly higher than that of the injection group and the control group. In the treatment effect evaluation test, it was found that the survival rate of the phage group was higher than that of some antibiotic groups, and the same phenomenon was also found in the phage treatment of mice infected with *pseudomonas aeruginosa*, and the survival rate of the phage treatment was 25% higher than that of the antibiotic treatment (Koderi Valappil et al., 2021). In addition, we also found that there was no significant difference in feed efficiency between the bacteriophage group and the negative control group in the treatment test, and it was significantly higher than other antibiotic groups. The feed efficiency in the prevention test was not only higher than that in the other antibiotic groups, but also higher than that in the negative control group, suggesting that phages may have a mechanism that can promote animal weight gain.

In conclusion, we isolated two novel virulent phages, C-3 and G21-7, with high-efficient lysis activity and a wide lysis spectrum. Phage C-3 and G21-7 have good tolerance to extreme environments. Genomic analysis showed that phage C-3 and G21-7 were new species of *Saphexavirus* genus. In addition, phages does not carry any resistance genes and virulence factors. It can effectively prevent and treat the infection of APEC from geese, and also has an inhibitory effect on human and animal (cattle, sheep, camels and pigeon) *E. coli*-serogroups such as O157, O26, O145, O178, O103 and O104. Therefore, phage C-3 and G21-7 are promising as bacteriostatic agents for pathogenic *E. coli*.

## Data availability statement

The datasets presented in this study can be found in online repositories. The names of the repository/repositories and accession number(s) can be found in the article/Supplementary material.

## Ethics statement

The animal study was approved by Animal Welfare and Ethics Committee of Xinjiang Agricultural University, College of Veterinary Medicine, Xinjiang Agricultural University. The study was conducted in accordance with the local legislation and institutional requirements.

## Author contributions

TW: Investigation, Writing – original draft. LZ: Writing – original draft, Writing – review & editing. YZ: Data curation, Writing – review & editing. PT: Data curation, Writing – review & editing. WM: Writing – original draft. YW: Investigation, Writing – original draft. YL: Investigation, Writing – original draft. ZS: Methodology, Project administration, Writing – review & editing.

## Footnotes

^1^https://euromeatnews.com/Article-The-global-duck-and-goose-meat-consumption-will-grow-to-8-million-tonnes/3267

^2^https://proksee.ca/

^3^https://phagescope.deepomics.org/

^4^http://tools.magigene.com/h5-BioCloud-leading-tools/tool/phaTYP_2022?viewId=2c908b7b894d321001894d3dba350005

^5^https://github.com/KennthShang/PhaGCN

^6^http://tools.magigene.com/h5-BioCloud-leading-tools/tool/cherry_2022?viewId=2c908b7b894d321001894d3f10c90009

^7^https://gbkviz.streamlit.app/

^8^https://www.chiplot.online/tvbot.html

^9^https://www.ncbi.nlm.nih.gov/Structure/bwrpsb/bwrpsb.cgi

^10^https://meme-suite.org/meme/
