# Supplementary material for: Isolation and identification of specific Enterococcus faecalis phage C-3 and G21-7 against Avian pathogenic Escherichia coli and its application to one-day-old geese
Source: Front Microbiol. 2024 Jun 19;15:1385860. doi: 10.3389/fmicb.2024.1385860 (PMC11221357; doi:10.3389/fmicb.2024.1385860)
Supplement: Supplementary file 1 [file Table_1.docx]

Supplementary Material

Supplementary Table1 Escherichia coli isolates used to examine the host range of phage

| bacteria | source | Year | Sample | Serogroup | Drug-resistant phenotype | C-3 | G21-7 | C-3 and C-5 |
| --- | --- | --- | --- | --- | --- | --- | --- | --- |
| D29 | Cattle | 2020 | excrement | O157 | CAZ-CTX-STX | ﹢ | ﹢ | ﹢ |
| NG7 | Cattle | 2021 | Anal swab | O157 | CTX-AZM -STX-CAZ-CTX | - | - | - |
| J14 | Cattle | 2018 | Carcass swab | O157 | AMP-CTX-AZM-CN-SAM-AN-CIP-STR-PIP-STX | ﹢ | - | ﹢ |
| X23 | Cattle | 2020 | excrement | O157 | STX-CTX-CAZ-FEP | - | - | - |
| NG8 | Cattle | 2021 | Anal swab | O157 | STX-AZM-AN | ﹢ | - | ﹢ |
| 20290 | Cattle | 2020 | Anal swab | O157 | AMP-CTX-AZM-CN-TE-STX-CHL-STR-PIP | - | - | - |
| XC25 | Camel | 2019 | excrement | O157 | AMP-AMC-CTX-CAZ-AZM-CN-SAM-AN-TE-CHL-STR-PIP | - | - | - |
| XC8 | Camel | 2019 | excrement | O157 | CTX-CAZ-AZM-AN-PIP | - | - | - |
| A2-32 | Camel | 2021 | excrement | O157 | AMP-CTX-CAZ-FEP-AZM-CN-TE-STX-CHL-STR-PIP | ﹢ | ﹢ | ﹢ |
| Y5 | Sheep | 2021 | excrement | O157 | LEV-CIP-TE-STX-STR-PIP | - | - | - |
| Q9 | Cattle | 2018 | Carcass swab | O26 | CTX-CAZ-TZP-AZM-PIP | ﹢ | - | ﹢ |
| 20342 | Cattle | 2020 | Anal swab | O26 | TE-STX-CHL-STR | - | ﹢ | ﹢ |
| 2268 | Cattle | 2020 | excrement | O26 | CHL-GEN-STR-STX-TE-TZP | ﹢ | ﹢ | ﹢ |
| T15 | Cattle | 2018 | Carcass swab | O26 | AMP-AMC-CTX-CAZ-FEP-TZP-AZM-CN-AN-LEV-TE-STX-CHL-PB-STR | - | - | - |
| L10 | Camel | 2020 | excrement | O26 | AMP-AMC-CTX-CAZ-AZM-CN | ﹢ | ﹢ | ﹢ |
| L6 | Camel | 2020 | excrement | O26 | CTX-CAZ-TZP-SAM-AN-STR | - | ﹢ | ﹢ |
| C2-11 | Camel | 2021 | excrement | O26 | AMP-CTX-CAZ-AZM-CN-TE-STX-CHL-STR-PIP | ﹢ | ﹢ | ﹢ |
| XC25 | Camel | 2019 | excrement | O26 | AMP-AMC-CTX-CAZ-AZM-CN-SAM-AN-TE-CHL-STR-PIP | - | ﹢ | ﹢ |
| XC13 | Camel | 2019 | excrement | O26 | CTX-CAZ-TZP-AZM-PIP | ﹢ | ﹢ | ﹢ |
| A2-33 | Camel | 2021 | excrement | O26 | AMP-CTX-CAZ-AZM-CN-TE-STX-CHL-STR-PIP | - | - | - |
| L14 | Camel | 2020 | excrement | O26 | CN-SAM-AN-TE-CHL-STR-PIP | ﹢ | ﹢ | ﹢ |
| YS18-2 | Sheep | 2019 | Anal swab | O26 | TZP-AMC-CHL | - | ﹢ | ﹢ |
| YS25-1 | Sheep | 2019 | Anal swab | O26 | TZP-PB-AMC-CAZ-SAM | ﹢ | ﹢ | ﹢ |
| W4 | pigeon | 2023 | Anal swab | O26 | GEN-AMP-CHL-AMI-LEV-STX-CIP-AMC | - | - | - |
| 272-2 | Human | 2019 | excrement | O26 | CHL-STR-TE | - | - | - |
| 324-1 | Human | 2019 | excrement | O26 | AMP-AZM-CAZ-CHL-CTX-FEP-GEN-PIP-STR-STX-TE | ﹢ | - | ﹢ |
| 360-1 | Human | 2019 | excrement | O26 | AMP | - | ﹢ | ﹢ |
| 272-6 | Human | 2019 | excrement | O26 | GEN-PIP-STR-STX-TE | ﹢ | ﹢ | ﹢ |
| N63 | Cattle | 2021 | excrement | O145 | TE | ﹢ | ﹢ | ﹢ |
| N140 | Cattle | 2021 | excrement | O145 | AMI-AMP-AZM-CAZ-CHL-CTX-GEN-PIP-SAM-STR-STX-TE | - | ﹢ | ﹢ |
| N234 | Cattle | 2021 | excrement | O145 | AMC-AMI-AMP-AZM-CAZ-CHL-CTX-PIP-STR-STX-TE | - | - | - |
| 12—5 | Camel | 2019 | Anal swab | O145 | AMP-AMC-CTX-CAZ-TZP-AZM-SAM-TE-STX-STR-PIP | ﹢ | - | ﹢ |
| 10—3 | Camel | 2019 | Anal swab | O145 | CTX-PB-PIP-CTX-CAZ-FEP-AZM-SAM-STX | ﹢ | ﹢ | ﹢ |
| B32g-4 | Camel | 2019 | Anal swab | O145 | AN-AZM-SAM-STX | ﹢ | ﹢ | ﹢ |
| B42g-2 | Camel | 2019 | Anal swab | O145 | AN-PIP | ﹢ | - | ﹢ |
| B34g-2 | Camel | 2019 | Anal swab | O145 | AZM-STR-PIP | - | - | - |
| G2-4 | Camel | 2019 | Anal swab | O145 | AZM-PIP-CTX-CAZ | - | ﹢ | ﹢ |
| 5—1 | Camel | 2019 | Anal swab | O145 | FEP-AZM-SAM-STX | ﹢ | - | ﹢ |
| 35—2 | Sheep | 2019 | Anal swab | O145 | AMP-PB | - | ﹢ | ﹢ |
| 3-20-3 | pigeon | 2023 | Anal swab | O145 | TE-CHL-AMI-LEV | - | - | - |
| 2--12--1 | pigeon | 2023 | Anal swab | O145 | AMI-LEV-FEP-TE-STR | - | ﹢ | ﹢ |
| 2--14--2 | pigeon | 2023 | Anal swab | O145 | GEN-AMP-CHL-AMI | ﹢ | ﹢ | ﹢ |
| 3--31--3 | pigeon | 2023 | Anal swab | O145 | TE-STR-STX-CIP-AMC | ﹢ | - | ﹢ |
| 3--40--1 | pigeon | 2023 | Anal swab | O145 | AMI-LEV-FEP-TE | - | - | - |
| 3--2--1 | pigeon | 2023 | Anal swab | O145 | GEN-AMP-CHL-AMI-LEV-FEP | ﹢ | - | ﹢ |
| 2--9--1 | pigeon | 2023 | Anal swab | O145 | STR-STX-CIP-AMC | - | ﹢ | ﹢ |
| 3--32--2 | pigeon | 2023 | Anal swab | O145 | AMP-CHL-AMI-LEV-FEP-TE | ﹢ | ﹢ | ﹢ |
| 3--12--4 | pigeon | 2023 | Anal swab | O145 | AMI-LEV-TE-STR | ﹢ | - | ﹢ |
| 2--10--3 | pigeon | 2023 | Anal swab | O145 | CIP-AMC-GEN | - | - | - |
| C5-3-2 | Camel | 2019 | excrement | O178 | AMP-CTX-CAZ-FEP-AZM-CN | - | ﹢ | ﹢ |
| B41g-1 | Camel | 2019 | Anal swab | O178 | CTX-PB-PIP-CTX-CAZ-FEP-AZM-SAM-STX | - | ﹢ | ﹢ |
| ZG5-1 | Sheep | 2019 | Anal swab | O178 | AMP-CAZ-SAM-PIP-TZP-PB | - | - | - |
| YS13-1 | Sheep | 2019 | Anal swab | O178 | SAM-PIP-TZP-PB | ﹢ | - | ﹢ |
| YS10-2 | Sheep | 2019 | Anal swab | O178 | AMP-CAZ-SAM-PIP | - | ﹢ | ﹢ |
| YS111-2 | Sheep | 2019 | Carcass swab | O178 | AZM-CAZ-CTX-TZP-PB | ﹢ | ﹢ | ﹢ |
| P21 | pigeon | 2023 | excrement | O178 | GEN-AMP-CHL-AMI-LEV | - | - | - |
| W6 | pigeon | 2023 | excrement | O103 | GEN-AMP-CHL-AMI-LEV-FEP-TE-STR-STX-CIP-AMC | - | ﹢ | ﹢ |
| N131 | Cattle | 2020 | excrement | O104 | CHL-STR-STX-TE | - | ﹢ | ﹢ |
| N164 | Cattle | 2020 | Anal swab | O104 | CHL-STR-STX-TE | ﹢ | ﹢ | ﹢ |
| ZG1-4 | Sheep | 2019 | Anal swab | O104 | TZP | ﹢ | ﹢ | ﹢ |
| YR12-1 | Sheep | 2019 | Carcass swab | O104 | AZM-CAZ-CTX | ﹢ | ﹢ | ﹢ |
| H1-4 | goose | 2023 | excrement | O1 | PIP-STR-PB-STX-TE-CIP-LEV-AMI-SAM-AZM-TZP-FEP-CAZ-CTX-AMC-AMP | ﹢ | ﹢ | ﹢ |
| Y-1 | goose | 2023 | excrement | O2 | STR-PB-CHL-STX-TE-CIP-LEV-AMI-SAM-TZP-FEP-CAZ-CTX-AMP | ﹢ | ﹢ | ﹢ |
| Y-9 | goose | 2023 | excrement | O2 | PIP-STR-CHL-TE-AZM-TZP-CAZ-CTX-AMP | ﹢ | ﹢ | ﹢ |
| H1-3 | goose | 2023 | excrement | O18 | PIP-PB-STX-TE-CIP | ﹢ | ﹢ | ﹢ |
| H1-5 | goose | 2023 | excrement | O18 | PIP-PB-STX-TE-CIP-LEV-AMI-AZM | - | - | - |
| H1-7 | goose | 2023 | excrement | O18 | STR-PB-CHL-STX-TE-CIP-LEV-SAM-CAZ-CTX-AMP-CN | ﹢ | ﹢ | ﹢ |
| Y-4 | goose | 2023 | excrement | O18 | PIP-STR-PB-STX-TE-CIP-LEV-AZM-FEP-CAZ-CTX-AMP | - | - | - |
| Y-8 | goose | 2023 | excrement | O18 | PIP-STR-PB-STX-TE-CIP-LEV-AZM-SAM-CAZ-CTX-AMP | ﹢ | ﹢ | ﹢ |
| Y-2 | goose | 2023 | excrement | O78 | PIP-PB-TE-CIP-LEV-CAZ | - | - | - |
| Y-5 | goose | 2023 | excrement | O78 | PIP-PB-TE-CIP-LEV-CAZ | ﹢ | ﹢ | ﹢ |
| Y-10 | goose | 2023 | excrement | O78 | PIP-PB-TE-CIP-LEV-CAZ | ﹢ | ﹢ | ﹢ |

Note: CAZ, ceftazidime; CTX, cefotaxime; AMP, ampicillin; LEV, levofloxacin; TE, tetracycline; CHL, chloramphenicol; CN, Penicillin; TZP, piperacillin-tazobactam; GEN, gentamicin; AMI, amikacin; AMC, amoxicillin-clavulanate; AN, Amoxicillin; AZM, Azithromycin; FEP, cefepime; STR, streptomycin; CIP, ciprofloxacin; SAM, ampicillin-sulbactam; PIP, piperacillin; PB, polymyxin B; STX, trimethoprim-sulfamethoxazole. “+” means clear lytic spots or plaques, “-” means no lytic spots or plaques.
