# Supplementary material for: Isolation and identification of specific Enterococcus faecalis phage C-3 and G21-7 against Avian pathogenic Escherichia coli and its application to one-day-old geese
Source: Front Microbiol. 2024 Jun 19;15:1385860. doi: 10.3389/fmicb.2024.1385860 (PMC11221357; doi:10.3389/fmicb.2024.1385860)
Supplement: Supplementary file 2 [file Table_2.docx]

Supplementary Material

Supplementary Table2 bacteriophage cross infection test with different serum groups of APEC as host bacteria

| Serogrope | O1 | O2 | | O18 | | | O78 | |
| --- | --- | --- | --- | --- | --- | --- | --- | --- |
| Name | H1-4 | Y-1 | Y-9 | H1-3 | H1-5 | Y-4 | Y-2 | Y-5 |
| C-3 | ﹢ | ﹢ | ﹢ | ﹢ | ﹢ | ﹢ | ﹢ | ﹢ |
| C-4 | ﹢ | - | - | - | - | - | - | - |
| C-5 | ﹢ | - | ﹢ | - | - | ﹢ | - | - |
| G3-1 | - | ﹢ | - | - | - | - | - | ﹢ |
| G3-2 | ﹢ | ﹢ | ﹢ | - | - | ﹢ | ﹢ | ﹢ |
| G4-4 | ﹢ | ﹢ | ﹢ | ﹢ | ﹢ | ﹢ | ﹢ | - |
| G4-5 | - | - | - | - | - | ﹢ | - | ﹢ |
| G21-7 | ﹢ | ﹢ | ﹢ | ﹢ | ﹢ | ﹢ | ﹢ | ﹢ |

Note: “+”means clear lytic spots or plaques, “-”means no lytic spots or plaques.
