# Supplementary material for: Isolation and identification of specific Enterococcus faecalis phage C-3 and G21-7 against Avian pathogenic Escherichia coli and its application to one-day-old geese
Source: Front Microbiol. 2024 Jun 19;15:1385860. doi: 10.3389/fmicb.2024.1385860 (PMC11221357; doi:10.3389/fmicb.2024.1385860)
Supplement: Supplementary file 3 [file Table_3.docx]

Supplementary Material

Supplementary Table3 The optimal multiplicity of infection (MOI)

| Phage name | Bacterial Concentration  (CFU·mL^-1^) | phage concentration  (PFU·mL^-1^) | MOI | phage titer Repeat 1  (PFU·mL^-1^) | phage titer Repeat 2  (PFU·mL^-1^) | phage titer Repeat 3  (PFU·mL^-1^) |
| --- | --- | --- | --- | --- | --- | --- |
| C-3 | 1.1 × 10^10^ | 1.1 × 10^8^ | 0.01 | 7.2 × 10^8^ | 6.1 × 10^8^ | 8.3 × 10^8^ |
|  | 1.1 × 10^10^ | 1.1 × 10^9^ | 0.1 | 3.5 × 10^9^ | 4.7 × 10^9^ | 2.3 × 10^9^ |
|  | 1.1 × 10^10^ | 1.1 × 10^10^ | 1 | 1.6 × 10^11^ | 2.5 × 10^11^ | 7.0 × 10^10^ |
|  | 1.1 × 10^10^ | 1.1 × 10^11^ | 10 | 6.4 × 10^11^ | 3.9 × 10^11^ | 8.9 × 10^11^ |
|  | 1.1 × 10^10^ | 1.1 × 10^12^ | 100 | 4.5 × 10^9^ | 6.3 × 10^9^ | 2.7 × 10^9^ |
|  | 1.1 × 10^10^ | 1.1 × 10^13^ | 1000 | 8.9 × 10^7^ | 6.7 × 10^7^ | 11.1 × 10^7^ |
| G21-7 | 3.8 × 10^10^ | 3.8 × 10^8^ | 0.01 | 1.2 × 10^10^ | 1.6 × 10^10^ | 8.0 × 10^9^ |
|  | 3.8 × 10^10^ | 3.8 × 10^9^ | 0.1 | 5.3 × 10^10^ | 8.2 × 10^10^ | 2.4 × 10^10^ |
|  | 3.8 × 10^10^ | 3.8 × 10^10^ | 1 | 7.3 × 10^12^ | 5.3 × 10^12^ | 9.3 × 10^12^ |
|  | 3.8 × 10^10^ | 3.8 × 10^11^ | 10 | 6.2 × 10^11^ | 4.5 × 10^11^ | 7.9 × 10^11^ |
|  | 3.8 × 10^10^ | 3.8 × 10^12^ | 100 | 8.9 × 10^11^ | 10.8 × 10^11^ | 7.5 × 10^11^ |
|  | 3.8 × 10^10^ | 3.8 × 10^13^ | 1000 | 4.3 × 10^9^ | 2.1 × 10^9^ | 6.5 × 10^9^ |
