# Supplementary material for: Isolation and identification of specific Enterococcus faecalis phage C-3 and G21-7 against Avian pathogenic Escherichia coli and its application to one-day-old geese
Source: Front Microbiol. 2024 Jun 19;15:1385860. doi: 10.3389/fmicb.2024.1385860 (PMC11221357; doi:10.3389/fmicb.2024.1385860)
Supplement: Supplementary file 4 [file Table_4.docx]

Supplementary Material

Supplementary Table4 One-step experiments

| Phage name | C-3 (PFU·mL^-1^) | | | G21-7 (PFU·mL^-1^) | | |
| --- | --- | --- | --- | --- | --- | --- |
| Time (min) | Repeat 1 | Repeat 2 | Repeat 3 | Repeat 1 | Repeat 2 | Repeat 3 |
| 10 | 1.2×10^6^ | 0.7×10^6^ | 1.7×10^6^ | 1.22×10^7^ | 0.9×10^7^ | 1.54×10^7^ |
| 20 | 1.03×10^7^ | 0.9×10^7^ | 1.16×10^7^ | 1.03×10^9^ | 0.6×10^9^ | 1.46×10^9^ |
| 30 | 8.0×10^7^ | 7.6×10^7^ | 8.7×10^7^ | 8.1×10^8^ | 7.7×10^8^ | 8.2×10^8^ |
| 40 | 8.2×10^8^ | 6.8×10^8^ | 9.3×10^8^ | 8.4×10^9^ | 6.3×10^9^ | 9.3×10^9^ |
| 50 | 8.1×10^9^ | 6.5×10^9^ | 9.7×10^9^ | 2.8×10^10^ | 1.5×10^10^ | 4.7×10^10^ |
| 60 | 3.9×10^10^ | 2.3×10^10^ | 5.5×10^10^ | 2.7×10^11^ | 2.3×10^11^ | 4.0×10^11^ |
| 70 | 3.5×10^11^ | 2.1×10^11^ | 4.9×10^11^ | 3.0×10^12^ | 2.1×10^12^ | 3.9×10^12^ |
| 80 | 3.1×10^12^ | 1.8×10^12^ | 4.4×10^12^ | 6.0×10^13^ | 3.9×10^13^ | 8.1×10^13^ |
| 90 | 2.8×10^11^ | 0.9×10^11^ | 4.7×10^11^ | 4.0×10^13^ | 1.8×10^13^ | 6.2×10^13^ |
| 100 | 7.2×10^10^ | 5.8×10^10^ | 8.6×10^10^ | 1.0×10^12^ | 0.8×10^12^ | 1.2×10^12^ |
