# Supplementary material for: Isolation and identification of specific Enterococcus faecalis phage C-3 and G21-7 against Avian pathogenic Escherichia coli and its application to one-day-old geese
Source: Front Microbiol. 2024 Jun 19;15:1385860. doi: 10.3389/fmicb.2024.1385860 (PMC11221357; doi:10.3389/fmicb.2024.1385860)
Supplement: Supplementary file 5 [file Table_5.docx]

Supplementary Material

Supplementary Table5 Temperature sensitivity test

| Phage name | C-3 (PFU·mL^-1^) | | | | | | G21-7 (PFU·mL^-1^) | | | | | |
| --- | --- | --- | --- | --- | --- | --- | --- | --- | --- | --- | --- | --- |
| Temperature  (℃) | 30min | | | 60min | | | 30min | | | 60min | | |
|  | Repeat 1 | Repeat 2 | Repeat 3 | Repeat 1 | Repeat 2 | Repeat 3 | Repeat 1 | Repeat 2 | Repeat 3 | Repeat 1 | Repeat 2 | Repeat 3 |
| 40 | 1.5×10^11^ | 1.7×10^11^ | 1.6×10^11^ | 0.8×10^11^ | 0.5×10^11^ | 1.1×10^11^ | 6.5×10^11^ | 5.4×10^11^ | 7.9×10^11^ | 6.5×10^11^ | 5.4×10^11^ | 7.9×10^11^ |
| 50 | 2.3×10^12^ | 1.1×10^12^ | 2.9×10^12^ | 3.2×10^6^ | 5×10^6^ | 1.4×10^6^ | 4.8×10^13^ | 4.1×10^13^ | 5.5×10^13^ | 4.8×10^13^ | 4.1×10^13^ | 5.5×10^13^ |
| 60 | 4.2×10^9^ | 2.8×10^9^ | 5.6×10^9^ | 4.2×10^8^ | 1.9×10^8^ | 6.5×10^8^ | 5.7×10^13^ | 4.6×10^13^ | 7.1×10^13^ | 5.7×10^13^ | 4.6×10^13^ | 7.1×10^13^ |
| 70 | 1.8×10^6^ | 0.4×10^6^ | 2.6×10^6^ | 3.6×10^6^ | 1.7×10^6^ | 4.3×10^6^ | 0 | 0 | 0 | 0 | 0 | 0 |
| 80 | 0 | 0 | 0 | 0 | 0 | 0 | 0 | 0 | 0 | 0 | 0 | 0 |
