# Supplementary material for: Isolation and identification of specific Enterococcus faecalis phage C-3 and G21-7 against Avian pathogenic Escherichia coli and its application to one-day-old geese
Source: Front Microbiol. 2024 Jun 19;15:1385860. doi: 10.3389/fmicb.2024.1385860 (PMC11221357; doi:10.3389/fmicb.2024.1385860)
Supplement: Supplementary file 6 [file Table_6.docx]

Supplementary Material

Supplementary Table6 PH sensitivity test

| Phage name | C-3 (PFU·mL^-1^) | | | G21-7 (PFU·mL^-1^) | | |
| --- | --- | --- | --- | --- | --- | --- |
| PH | Repeat 1 | Repeat 2 | Repeat 3 | Repeat 1 | Repeat 2 | Repeat 3 |
| 4 | 0 | 0 | 0 | 3.2×10^4^ | 2.8×10^4^ | 3.6×10^4^ |
| 5 | 1.5×10^6^ | 1.3×10^6^ | 2.0×10^6^ | 3.8×10^5^ | 3.9×10^5^ | 5.8×10^5^ |
| 6 | 3.3×10^8^ | 2.6×10^8^ | 3.7×10^8^ | 2.2×10^8^ | 1.2×10^8^ | 2.9×10^8^ |
| 7 | 3.5×10^9^ | 2.4×10^9^ | 4.6×10^9^ | 7.7×10^9^ | 6.0×10^9^ | 9.1×10^9^ |
| 8 | 7.8×10^10^ | 6.5×10^10^ | 8.2×10^10^ | 5.7×10^10^ | 3.8×10^10^ | 6.7×10^10^ |
| 9 | 8.1×10^10^ | 8.3×10^10^ | 9.1×10^10^ | 3.6×10^10^ | 3.2×10^10^ | 4.3×10^10^ |
| 10 | 5.4×10^11^ | 4.5×10^11^ | 8.7×10^11^ | 5.3×10^11^ | 4.3×10^11^ | 5.2×10^11^ |
| 11 | 6.5×10^10^ | 4.2×10^10^ | 6.1×10^10^ | 10.5×10^10^ | 7.0×10^10^ | 11.3×10^10^ |
| 12 | 0 | 0 | 0 | 0 | 0 | 0 |
