# Supplementary material for: Isolation and identification of specific Enterococcus faecalis phage C-3 and G21-7 against Avian pathogenic Escherichia coli and its application to one-day-old geese
Source: Front Microbiol. 2024 Jun 19;15:1385860. doi: 10.3389/fmicb.2024.1385860 (PMC11221357; doi:10.3389/fmicb.2024.1385860)
Supplement: Supplementary file 9 [file Table_9.docx]

Supplementary Material

Supplementary Table9 Safety test of phage mixtures In *vivo*

| Group (Number) | | Oral (n=50) | | | | | Intraperitoneal injection (n=50) | | | | | Control group (n=50) | | | | |
| --- | --- | --- | --- | --- | --- | --- | --- | --- | --- | --- | --- | --- | --- | --- | --- | --- |
|  |  | 1 | 2 | 3 | 4 | 5 | 1 | 2 | 3 | 4 | 5 | 1 | 2 | 3 | 4 | 5 |
| Weekly weight  (Kg) | Day 0 | 0.70 | 0.70 | 0.70 | 0.70 | 0.70 | 0.70 | 0.70 | 0.70 | 0.70 | 0.70 | 0.70 | 0.70 | 0.70 | 0.70 | 0.70 |
|  | Day 7 | 1.50 | 1.50 | 1.60 | 1.40 | 1.50 | 1.60 | 1.40 | 1.50 | 1.40 | 1.44 | 1.50 | 1.40 | 1.50 | 1.60 | 1.20 |
|  | Day 14 | 3.00 | 2.90 | 2.80 | 3.10 | 3.20 | 3.00 | 3.10 | 2.80 | 3.20 | 2.32 | 3.00 | 2.90 | 2.80 | 2.70 | 1.82 |
|  | Day 21 | 6.40 | 6.00 | 6.10 | 5.90 | 5.60 | 5.90 | 5.60 | 6.00 | 5.80 | 4.64 | 5.60 | 5.40 | 5.70 | 5.50 | 4.06 |
| Daily feed intake  (Kg) | Day 1 | 0.16 | 0.14 | 0.16 | 0.14 | 0.15 | 0.16 | 0.17 | 0.16 | 0.15 | 0.16 | 0.15 | 0.15 | 0.15 | 0.15 | 0.14 |
|  | Day 2 | 0.18 | 0.17 | 0.18 | 0.17 | 0.18 | 0.19 | 0.19 | 0.19 | 0.19 | 0.19 | 0.17 | 0.18 | 0.18 | 0.18 | 0.17 |
|  | Day 3 | 0.23 | 0.23 | 0.24 | 0.22 | 0.23 | 0.24 | 0.24 | 0.24 | 0.21 | 0.24 | 0.23 | 0.22 | 0.23 | 0.23 | 0.23 |
|  | Day 4 | 0.25 | 0.25 | 0.25 | 0.24 | 0.27 | 0.27 | 0.27 | 0.27 | 0.26 | 0.27 | 0.25 | 0.25 | 0.25 | 0.26 | 0.24 |
|  | Day 5 | 0.27 | 0.27 | 0.27 | 0.26 | 0.27 | 0.29 | 0.28 | 0.29 | 0.28 | 0.29 | 0.27 | 0.27 | 0.27 | 0.28 | 0.25 |
|  | Day 6 | 0.29 | 0.29 | 0.29 | 0.29 | 0.29 | 0.31 | 0.31 | 0.31 | 0.30 | 0.32 | 0.29 | 0.28 | 0.29 | 0.31 | 0.27 |
|  | Day 7 | 0.31 | 0.32 | 0.32 | 0.31 | 0.31 | 0.34 | 0.34 | 0.34 | 0.33 | 0.34 | 0.31 | 0.31 | 0.33 | 0.32 | 0.30 |
|  | Day 8 | 0.28 | 0.28 | 0.28 | 0.28 | 0.28 | 0.29 | 0.28 | 0.29 | 0.29 | 0.28 | 0.28 | 0.28 | 0.28 | 0.26 | 0.25 |
|  | Day 9 | 0.33 | 0.33 | 0.34 | 0.33 | 0.34 | 0.34 | 0.32 | 0.34 | 0.33 | 0.34 | 0.43 | 0.31 | 0.32 | 0.32 | 0.20 |
|  | Day 10 | 0.43 | 0.43 | 0.43 | 0.43 | 0.44 | 0.45 | 0.41 | 0.44 | 0.43 | 0.44 | 0.50 | 0.42 | 0.41 | 0.42 | 0.29 |
|  | Day 11 | 0.47 | 0.48 | 0.48 | 0.47 | 0.49 | 0.49 | 0.46 | 0.50 | 0.47 | 0.48 | 0.56 | 0.46 | 0.46 | 0.48 | 0.31 |
|  | Day 12 | 0.50 | 0.51 | 0.50 | 0.50 | 0.50 | 0.64 | 0.41 | 0.51 | 0.49 | 0.49 | 0.60 | 0.49 | 0.48 | 0.47 | 0.35 |
|  | Day 13 | 0.50 | 0.51 | 0.50 | 0.50 | 0.50 | 0.68 | 0.48 | 0.58 | 0.45 | 0.56 | 0.60 | 0.52 | 0.57 | 0.50 | 0.41 |
|  | Day 14 | 0.59 | 0.59 | 0.58 | 0.60 | 0.60 | 0.53 | 0.51 | 0.53 | 0.52 | 0.53 | 0.59 | 0.53 | 0.53 | 0.54 | 0.43 |
|  | Day 15 | 0.54 | 0.55 | 0.54 | 0.55 | 0.55 | 0.63 | 0.61 | 0.63 | 0.61 | 0.61 | 0.61 | 0.52 | 0.53 | 0.52 | 0.44 |
|  | Day 16 | 0.60 | 0.61 | 0.62 | 0.67 | 0.70 | 0.82 | 0.72 | 0.84 | 0.85 | 0.77 | 0.62 | 0.62 | 0.75 | 0.62 | 0.48 |
|  | Day 17 | 0.83 | 0.83 | 0.83 | 0.83 | 0.84 | 0.88 | 0.79 | 0.90 | 0.97 | 0.89 | 0.90 | 0.83 | 0.90 | 0.80 | 0.55 |
|  | Day 18 | 0.92 | 0.92 | 0.92 | 0.92 | 0.92 | 0.94 | 0.90 | 0.94 | 0.91 | 0.99 | 0.91 | 0.92 | 1.07 | 0.90 | 0.61 |
|  | Day 19 | 0.98 | 0.98 | 0.97 | 0.97 | 0.95 | 1.01 | 0.98 | 1.02 | 1.05 | 1.02 | 1.03 | 0.88 | 1.02 | 0.99 | 0.69 |
|  | Day 20 | 1.07 | 1.06 | 1.05 | 1.05 | 1.04 | 1.10 | 1.09 | 1.12 | 1.12 | 1.08 | 1.02 | 1.09 | 1.05 | 1.03 | 0.87 |
|  | Day 21 | 1.16 | 1.15 | 1.14 | 1.14 | 1.13 | 1.16 | 1.08 | 1.25 | 1.21 | 0.80 | 1.22 | 1.20 | 1.12 | 1.04 | 0.91 |
| Weekly deaths | 1 week | 0 | | | | | 1 | | | | | 2 | | | | |
|  | 2 weeks | 0 | | | | | 1 | | | | | 1 | | | | |
|  | 3 weeks | 0 | | | | | 0 | | | | | 0 | | | | |
