# Supplementary material for: Isolation and identification of specific Enterococcus faecalis phage C-3 and G21-7 against Avian pathogenic Escherichia coli and its application to one-day-old geese
Source: Front Microbiol. 2024 Jun 19;15:1385860. doi: 10.3389/fmicb.2024.1385860 (PMC11221357; doi:10.3389/fmicb.2024.1385860)
Supplement: Supplementary file 11 [file Table_11.docx]

Supplementary Material

Supplementary Table11 Efficacy evaluation of bacteriophage preparations and antibiotics for prevention of APEC infection in *vivo*

| Group (Number) | | SHLHSP (n=50) | DHSP (n=50) | CSSSP (n=50) | FF (n=50) | TFSP (n=50) | Phage (n=50) | PC (n=50) | NC (n=32) |
| --- | --- | --- | --- | --- | --- | --- | --- | --- | --- |
| Weekly weight  (Kg) | Day 0 | 3.50 | 3.5 | 3.5 | 3.5 | 3.5 | 3.5 | 3.5 | 2.8 |
|  | Day 7 | 6 | 4.94 | 4.8 | 5.46 | 6.02 | 5.46 | 3.5 | 4.8 |
|  | Day 14 | 10.2 | 9.36 | 9.25 | 8.4 | 9.24 | 11.48 | 1.84 | 8.06 |
|  | Day 21 | 16.8 | 14.7 | 17.39 | 13 | 16.38 | 22.4 | 1.32 | 15.08 |
| Daily feed intake  (Kg) | Day 1 | 0.92 | 0.37 | 0.81 | 0.6 | 0.93 | 0.93 | 0.38 | 0.62 |
|  | Day 2 | 0.93 | 0.37 | 0.82 | 0.61 | 0.94 | 0.94 | 0.38 | 0.62 |
|  | Day 3 | 1.04 | 0.42 | 0.92 | 0.68 | 1.05 | 1.05 | 0.43 | 0.7 |
|  | Day 4 | 1.33 | 0.53 | 1.18 | 0.87 | 1.34 | 1.34 | 0.55 | 0.89 |
|  | Day 5 | 1.39 | 0.56 | 1.23 | 0.91 | 1.41 | 1.41 | 0.57 | 0.93 |
|  | Day 6 | 1.5 | 0.6 | 1.33 | 0.99 | 1.52 | 1.52 | 0.62 | 1.01 |
|  | Day 7 | 1.63 | 0.65 | 1.45 | 1.07 | 1.65 | 1.65 | 0.67 | 1.1 |
|  | Day 8 | 0.96 | 1.36 | 1.61 | 1.47 | 1.87 | 1.76 | 0.19 | 0.99 |
|  | Day 9 | 0.96 | 1.37 | 1.63 | 1.49 | 1.89 | 1.78 | 0.19 | 1 |
|  | Day 10 | 1.08 | 1.54 | 1.83 | 1.67 | 2.12 | 2 | 0.22 | 1.13 |
|  | Day 11 | 1.38 | 1.97 | 2.33 | 2.13 | 2.71 | 2.55 | 0.28 | 1.44 |
|  | Day 12 | 1.45 | 2.06 | 2.44 | 2.23 | 2.84 | 2.67 | 0.29 | 1.51 |
|  | Day 13 | 1.57 | 2.23 | 2.64 | 2.41 | 3.07 | 2.89 | 0.31 | 1.63 |
|  | Day 14 | 1.7 | 2.42 | 2.87 | 2.62 | 3.34 | 3.14 | 0.34 | 1.77 |
|  | Day 15 | 1.31 | 1.61 | 1.89 | 1.43 | 1.82 | 1.28 | 0.23 | 1.95 |
|  | Day 16 | 1.32 | 1.62 | 1.91 | 1.44 | 1.83 | 1.29 | 0.23 | 1.96 |
|  | Day 17 | 1.48 | 1.82 | 2.14 | 1.62 | 2.06 | 1.45 | 0.26 | 2.21 |
|  | Day 18 | 1.9 | 2.33 | 2.73 | 2.07 | 2.63 | 1.85 | 0.33 | 2.82 |
|  | Day 19 | 1.98 | 2.43 | 2.86 | 2.16 | 2.75 | 1.93 | 0.34 | 2.95 |
|  | Day 20 | 2.14 | 2.63 | 3.09 | 2.34 | 2.97 | 2.09 | 0.37 | 3.19 |
|  | Day 21 | 2.33 | 2.86 | 3.36 | 2.54 | 3.23 | 2.27 | 0.4 | 3.47 |
| Weekly deaths | 1 week | 6 | 8 | 6 | 3 | 2 | 3 | 25 | 0 |
|  | 2 weeks | 6 | 2 | 3 | 3 | 1 | 1 | 17 | 2 |
|  | 3 weeks | 4 | 0 | 0 | 0 | 0 | 2 | 2 | 0 |

Note: TFSP, Tiamulin Fumarate Soluble Powder (Veterinary Drug Character (VDC) 020033008, Ringpu, China); FF, Fubennikao Fen (VDC 010122539, Aether Centre (Beijing) BIOLOGY, China); CSSSP, Compound Sulfamonomethoxine Sodium Soluble Powder (VDC 040266233, SHANXI YI KANG ANIMAL'S PHARMACEUTCAL, China); DHSP, Doxycycline Hyclate Soluble Powder (VDC 010126011, Aether Centre (Beijing) BIOLOGY, China); SHLHSP, Spectinomycin Hydrochloride and Lincomycin Hydrochloride Soluble Powder (VDC 20031339, ringpu, China); PC, positive control; NC, negative control.
