# Supplementary material for: Isolation and identification of specific Enterococcus faecalis phage C-3 and G21-7 against Avian pathogenic Escherichia coli and its application to one-day-old geese
Source: Front Microbiol. 2024 Jun 19;15:1385860. doi: 10.3389/fmicb.2024.1385860 (PMC11221357; doi:10.3389/fmicb.2024.1385860)
Supplement: Supplementary file 12 [file Table_12.docx]

Supplementary Material

Supplementary Table12 Multiple comparisons of survival analysis

| survival analysis | Log-rank (Mantel-Cox) test | | | Hazard Ratio (logrank) | |
| --- | --- | --- | --- | --- | --- |
|  | P value | Chi-square | Df | Ratio | 95% CI of ratio |
| **Safety test** | | | | | |
| Oral vs. Intraperitoneal injection | 0.1552 (ns) | 2.020 | 1 | Undefined | Undefined |
| Oral vs. Control group | 0.0801 (ns) | 3.062 | 1 | Undefined | Undefined |
| Intraperitoneal injection vs. Control group | 0.6639 (ns) | 0.1888 | 1 | 0.6749 | 0.1169 to 3.896 |
| **Treatment test** | | | | | |
| Phage vs. TFSP | 0.0126 (*) | 6.231 | 1 | 0.5198 | 0.3051 to 0.8855 |
| Phage vs. FF | 0.0005 (***) | 12.21 | 1 | 0.4164 | 0.2465 to 0.7033 |
| Phage vs. CSSSP | 0.6183 (ns) | 0.2482 | 1 | 0.8677 | 0.4898 to 1.537 |
| Phage vs. DHSP | 0.3783 (ns) | 0.7762 | 1 | 0.7800 | 0.4428 to 1.374 |
| Phage vs. SHLHSP | 0.0005 (***) | 11.97 | 1 | 0.4408 | 0.2601 to 0.7469 |
| Phage vs. PC | <0.0001 (****) | 23.50 | 1 | 0.3178 | 0.1938 to 0.5214 |
| Phage vs. NC | 0.0003 (***) | 12.98 | 1 | 8.651 | 3.864 to 19.37 |
| **Prevention test** | | | | | |
| Phage vs. TFSP | 0.3114 (ns) | 1.025 | 1 | 2.015 | 0.5454 to 7.442 |
| Phage vs. FF | 0.9777 (ns) | 0.0007814 | 1 | 0.9841 | 0.3174 to 3.051 |
| Phage vs. CSSSP | 0.3833 (ns) | 0.7600 | 1 | 0.6360 | 0.2311 to 1.750 |
| Phage vs. DHSP | 0.2459 (ns) | 1.346 | 1 | 0.5554 | 0.2083 to 1.481 |
| Phage vs. SHLHSP | 0.0155 (*) | 5.857 | 1 | 0.3346 | 0.1449 to 0.7729 |
| Phage vs. PC | <0.0001 (****) | 66.15 | 1 | 0.07497 | 0.04200 to 0.1338 |
| Phage vs. NC | 0.3847 (ns) | 0.7556 | 1 | 2.000 | 0.4862 to 8.230 |

Note: TFSP, Tiamulin Fumarate Soluble Powder (Veterinary Drug Character (VDC) 020033008, Ringpu, China); FF, Fubennikao Fen (VDC 010122539, Aether Centre (Beijing) Biology, China); CSSSP, Compound Sulfamonomethoxine Sodium Soluble Powder (VDC 040266233, Shanxi Yi Kang Animal'S Pharmaceutcal, China); DHSP, Doxycycline Hyclate Soluble Powder (VDC 010126011, Aether Centre (Beijing) Biology, China); SHLHSP, Spectinomycin Hydrochloride and Lincomycin Hydrochloride Soluble Powder (VDC 20031339, Ringpu, China); PC, positive control; NC, negative control; Df, degree of freedom; 95% CI, 95% confidence interval; ns, not significant; “*”, *p* < 0.05; “**”, *p* < 0.01; “***”, *p* < 0.001; “****”, *p* < 0.0001.
