# Supplementary material for: Isolation and identification of specific Enterococcus faecalis phage C-3 and G21-7 against Avian pathogenic Escherichia coli and its application to one-day-old geese
Source: Front Microbiol. 2024 Jun 19;15:1385860. doi: 10.3389/fmicb.2024.1385860 (PMC11221357; doi:10.3389/fmicb.2024.1385860)
Supplement: Supplementary file 13 [file Table_13.docx]

Supplementary Material

Supplementary Table 13 *Enterococcus faecalis* isolates used to examine the host range of phage

| bacteria | source | Year | Sample | C-3 | G21-7 | C-3 and C-5 |
| --- | --- | --- | --- | --- | --- | --- |
| G21 | Cattle | 2021 | excrement | - | - | - |
| G10 | Cattle | 2021 | excrement | - | - | - |
| G2 | Cattle | 2021 | excrement | - | - | - |
| 1-11 | Cattle | 2022 | excrement | - | - | - |
| G857 | Cattle | 2021 | excrement | - | - | - |
| G256 | Cattle | 2021 | excrement | - | - | - |
| G316 | Cattle | 2021 | excrement | - | - | - |
| G453 | Cattle | 2021 | excrement | - | - | - |
| G6 | Cattle | 2021 | excrement | - | - | - |
| DG341 | Cattle | 2023 | excrement | - | - | - |
| 1-17 | Cattle | 2022 | excrement | - | - | - |
| X30G20 | Cattle | 2023 | excrement | - | - | - |
| G14 | Cattle | 2021 | excrement | - | - | - |
| DG8 | Cattle | 2023 | excrement | - | - | - |
| G249 | Cattle | 2021 | excrement | - | - | - |
| G3 | Cattle | 2021 | excrement | - | - | - |
| G16 | Cattle | 2021 | excrement | - | - | - |
| G17 | Cattle | 2021 | excrement | - | - | - |
| DG1 | Cattle | 2019 | excrement | - | - | - |
| G1215 | Cattle | 2021 | excrement | - | - | - |
| DG11 | Cattle | 2020 | excrement | - | - | - |
| DG352 | Cattle | 2019 | excrement | - | - | - |
| DG6 | Cattle | 2019 | excrement | - | - | - |
| G1160 | Cattle | 2021 | excrement | - | - | - |
| 1-4 | Cattle | 2019 | excrement | - | - | - |
| G1DF | Cattle | 2021 | excrement | - | - | - |
| DG33 | Cattle | 2019 | excrement | - | - | - |
| G150 | Cattle | 2021 | excrement | - | - | - |
| 1-1 | Cattle | 2022 | excrement | - | - | - |
| G655 | Cattle | 2021 | excrement | - | - | - |
| G554 | Cattle | 2021 | excrement | - | - | - |
| G4-46 | Cattle | 2021 | excrement | - | - | - |
| G4 | Cattle | 2021 | excrement | - | - | - |
| 1-18 | Cattle | 2022 | excrement | - | - | - |
| DJ610 | Cattle | 2023 | excrement | - | - | - |
| X12G | Cattle | 2023 | excrement | - | - | - |
| G44 | Cattle | 2021 | excrement | - | - | - |
| DXG5 | Cattle | 2023 | excrement | - | - | - |
| G118 | Cattle | 2021 | excrement | - | - | - |
| G9 | Cattle | 2021 | excrement | - | - | - |
| 1-24 | Cattle | 2022 | excrement | - | - | - |
| G1547 | Cattle | 2021 | excrement | - | - | - |
| G19 | Cattle | 2021 | excrement | - | - | - |
| 1 | pigeon | 2021 | excrement | - | - | - |
| 2 | pigeon | 2021 | excrement | - | - | - |
| 3 | pigeon | 2021 | excrement | - | - | - |
| 4 | pigeon | 2021 | excrement | - | - | - |
| 5 | pigeon | 2021 | excrement | - | - | - |
| 6 | pigeon | 2021 | excrement | - | - | - |
| 7 | pigeon | 2021 | excrement | - | - | - |
| 8 | pigeon | 2021 | excrement | - | - | - |
| 10 | pigeon | 2021 | excrement | - | - | - |
| 11 | pigeon | 2021 | excrement | - | - | - |
| 12 | pigeon | 2021 | excrement | - | - | - |
| 16 | pigeon | 2021 | excrement | - | - | - |
| 17 | pigeon | 2021 | excrement |  |  |  |
| 18 | pigeon | 2021 | excrement | - | - | - |
| 20 | pigeon | 2021 | excrement | - | - | - |
| 21 | pigeon | 2021 | excrement | - | - | - |
| 24 | pigeon | 2021 | excrement | - | - | - |
| 25 | pigeon | 2021 | excrement | - | - | - |
| 28 | pigeon | 2021 | excrement | - | - | - |
| 34 | pigeon | 2021 | excrement | - | - | - |
| 35 | pigeon | 2021 | excrement | - | - | - |
| 37 | pigeon | 2021 | excrement | - | - | - |
| 39 | pigeon | 2021 | excrement | - | - | - |
| 41 | pigeon | 2021 | excrement | - | - | - |
| 42 | pigeon | 2021 | excrement | - | - | - |
| 43 | pigeon | 2021 | excrement | - | - | - |
| 83 | pigeon | 2021 | excrement | - | - | - |
| 111 | pigeon | 2021 | excrement | - | - | - |
| 116 | pigeon | 2021 | excrement | - | - | - |
| 142 | pigeon | 2021 | excrement | - | - | - |
| Wan-B16 | Camel | 2018 | excrement | - | - | - |
| A92 | Camel | 2020 | excrement | - | - | - |
| 1B | Camel | 2021 | excrement | - | - | - |
| WG9 | Camel | 2018 | excrement | - | - | - |
| FG4 | Camel | 2022 | excrement | - | - | - |
| HY9 | Camel | 2023 | excrement | - | - | - |
| HY15 | Camel | 2023 | excrement | - | - | - |
| A920 | Camel | 2020 | excrement | - | - | - |
| FG11 | Camel | 2022 | excrement | - | - | - |
| B22 | Camel | 2021 | excrement | - | - | - |
| FB16 | Camel | 2022 | excrement | - | - | - |
| 4-13 | Camel | 2020 | excrement | - | - | - |
| WY-9 | Camel | 2018 | excrement | - | - | - |
| AG13 | Camel | 2020 | excrement | - | - | - |
| G249 | Camel | 2019 | excrement | - | - | - |
| T3 | Camel | 2022 | excrement | - | - | - |
| FB19 | Camel | 2022 | excrement | - | - | - |
| AG10 | Camel | 2020 | excrement | - | - | - |
| H910 | Camel | 2023 | excrement | - | - | - |
| Y911 | Camel | 2021 | excrement | - | - | - |
| HG9 | Camel | 2023 | excrement | - | - | - |
| AB16 | Camel | 2020 | excrement | - | - | - |
| G20 | Camel | 2019 | excrement | - | - | - |
| Lung-L | Camel | 2023 | excrement | - | - | - |
| AG18 | Camel | 2020 | excrement | - | - | - |
| G11 | Camel | 2019 | excrement | - | - | - |
| Wan-B7 | Camel | 2018 | excrement | - | - | - |
| HY24 | Camel | 2023 | excrement | - | - | - |
| G8 | Camel | 2019 | excrement | - | - | - |
| AG9 | Camel | 2020 | excrement | - | - | - |
| b1 | Camel | 2019 | excrement | - | - | - |
| EL | Camel | 2022 | excrement | - | - | - |
| B23 | Camel | 2021 | excrement | - | - | - |
| B3 | Camel | 2021 | excrement | - | - | - |
| 1-bF | Camel | 2019 | excrement | - | - | - |
| Wan-B6 | Camel | 2018 | excrement | - | - | - |
| T1G9 | Camel | 2022 | excrement | - | - | - |
| FG2 | Camel | 2022 | excrement | - | - | - |
| B6 | Camel | 2021 | excrement | - | - | - |
| G6 | Camel | 2019 | excrement | - | - | - |
| WY17 | Camel | 2018 | excrement | - | - | - |
| T1G19 | Camel | 2022 | excrement | - | - | - |
| B8 | Camel | 2021 | excrement | - | - | - |
| AB14 | Camel | 2020 | excrement | - | - | - |
| A914 | Camel | 2020 | excrement | - | - | - |
| AB5 | Camel | 2020 | excrement | - | - | - |
| G8 | Camel | 2019 | excrement | - | - | - |
| T1G7 | Camel | 2022 | excrement | - | - | - |
| HG27 | Camel | 2023 | excrement | - | - | - |
| E1 | goose | 2023 | excrement | - | - | - |
| E2 | goose | 2023 | excrement | - | - | - |
| E3 | goose | 2023 | excrement | - | - | - |
| E4 | goose | 2023 | excrement | - | - | - |
| E5 | goose | 2023 | excrement | - | - | - |
| E5 | goose | 2023 | excrement | - | - | - |
| E6 | goose | 2023 | excrement | - | - | - |
| E7 | goose | 2023 | excrement | - | - | - |
| E8 | goose | 2023 | excrement | - | - | - |
| E9 | goose | 2023 | excrement | - | - | - |
| E10 | goose | 2023 | excrement | - | - | - |
| E11 | goose | 2023 | excrement | - | - | - |
| E12 | goose | 2023 | excrement | - | - | - |
| E13 | goose | 2023 | excrement | - | - | - |
| E14 | goose | 2023 | excrement | - | - | - |
| E15 | goose | 2023 | excrement | - | - | - |
| E16 | goose | 2023 | excrement | - | - | - |
| E17 | goose | 2023 | excrement | - | - | - |
| E18 | goose | 2023 | excrement | - | - | - |
| E19 | goose | 2023 | excrement | - | - | - |
| E20 | goose | 2023 | excrement | - | - | - |
| E22 | goose | 2023 | excrement | - | - | - |
| E23 | goose | 2023 | excrement |  |  |  |
| E24 | goose | 2023 | excrement | - | - | - |
| E26 | goose | 2023 | excrement | - | - | - |
| E27 | goose | 2023 | excrement | - | - | - |
| E28 | goose | 2023 | excrement | - | - | - |
| E31 | goose | 2023 | excrement | - | - | - |
| E35 | goose | 2023 | excrement | - | - | - |
| E37 | goose | 2023 | excrement | - | - | - |
| E38 | goose | 2023 | excrement | - | - | - |
| E39 | goose | 2023 | excrement | - | - | - |
| E40 | goose | 2023 | excrement | - | - | - |
| E41 | goose | 2023 | excrement | - | - | - |
| E42 | goose | 2023 | excrement | - | - | - |
| E44 | goose | 2023 | excrement | - | - | - |
| E45 | goose | 2023 | excrement | - | - | - |
| E46 | goose | 2023 | excrement | - | - | - |
| E47 | goose | 2023 | excrement | - | - | - |
| E48 | goose | 2023 | excrement | - | - | - |
| E49 | goose | 2023 | excrement | - | - | - |
| S19 | pig | 2021 | excrement | - | - | - |
| S20 | pig | 2021 | excrement | - | - | - |
| S21 | pig | 2021 | excrement | - | - | - |
| S23 | pig | 2021 | excrement | - | - | - |
| S24 | pig | 2021 | excrement | - | - | - |
| S26 | pig | 2021 | excrement | - | - | - |
| S27 | pig | 2021 | excrement | - | - | - |
| S28 | pig | 2021 | excrement | - | - | - |
| S29 | pig | 2021 | excrement | - | - | - |
| S33 | pig | 2021 | excrement | - | - | - |
| S34 | pig | 2021 | excrement | - | - | - |
| S36 | pig | 2021 | excrement | - | - | - |
| S37 | pig | 2021 | excrement | - | - | - |
| S38 | pig | 2021 | excrement | - | - | - |
| S42 | pig | 2021 | excrement | - | - | - |
| S52 | pig | 2021 | excrement | - | - | - |
| P7 | pig | 2020 | excrement | - | - | - |
| P10 | pig | 2020 | excrement | - | - | - |
| P18 | pig | 2020 | excrement | - | - | - |
| P25 | pig | 2020 | excrement | - | - | - |
| P26 | pig | 2020 | excrement | - | - | - |
| P29 | pig | 2020 | excrement | - | - | - |
| P30 | pig | 2020 | excrement | - | - | - |
| P31 | pig | 2020 | excrement | - | - | - |
| P34 | pig | 2020 | excrement | - | - | - |
| P37 | pig | 2020 | excrement | - | - | - |
| P44 | pig | 2020 | excrement | - | - | - |
| P54 | pig | 2020 | excrement | - | - | - |
| P56 | pig | 2020 | excrement | - | - | - |
| P58 | pig | 2020 | excrement | - | - | - |
| P59 | pig | 2020 | excrement | - | - | - |
| P60 | pig | 2020 | excrement | - | - | - |
| P68 | pig | 2020 | excrement | - | - | - |
| P75 | pig | 2020 | excrement | - | - | - |
| P86 | pig | 2020 | excrement | - | - | - |
| P94 | pig | 2020 | excrement | - | - | - |
| P101 | pig | 2020 | excrement | - | - | - |
| P105 | pig | 2020 | excrement | - | - | - |
| P109 | pig | 2020 | excrement | - | - | - |
| P112 | pig | 2020 | excrement | - | - | - |
| P113 | pig | 2020 | excrement | - | - | - |
| P127 | pig | 2020 | excrement | - | - | - |
| 3-1 | dog | 2022 | excrement | - | - | - |
| 11-1 | dog | 2022 | excrement | - | - | - |
| 21-1 | dog | 2022 | excrement | - | - | - |
| 22-1 | dog | 2022 | excrement | - | - | - |
| 24-5 | dog | 2022 | excrement | - | - | - |
| 26-1 | dog | 2022 | excrement | - | - | - |
| 26-3 | dog | 2022 | excrement | - | - | - |
| 27-1 | dog | 2022 | excrement | - | - | - |
| 27-3 | dog | 2022 | excrement | - | - | - |
| 28-2 | dog | 2022 | excrement | - | - | - |
| 29-1 | dog | 2022 | excrement | - | - | - |
| 30-1 | dog | 2022 | excrement | - | - | - |
| 31-1 | dog | 2022 | excrement | - | - | - |
| 34-2 | dog | 2022 | excrement | - | - | - |
| 35-1 | dog | 2022 | excrement | - | - | - |
| 37-3 | dog | 2022 | excrement | - | - | - |
| 38-1 | dog | 2022 | excrement | - | - | - |
| 39-4 | dog | 2022 | excrement | - | - | - |
